# Stabilization of CCDC102B by Loss of RACK1 Through the CMA Pathway Promotes Breast Cancer Metastasis *via* Activation of the NF-κB Pathway

**DOI:** 10.3389/fonc.2022.927358

**Published:** 2022-07-25

**Authors:** Jing Si, Rong Guo, Bingqiu Xiu, Weiru Chi, Qi Zhang, Jianjing Hou, Yonghui Su, Jiajian Chen, Jingyan Xue, Zhi-Ming Shao, Jiong Wu, Yayun Chi

**Affiliations:** ^1^ Department of Breast Surgery, Key Laboratory of Breast Cancer in Shanghai, Fudan University Shanghai Cancer Center, Shanghai, China; ^2^ Department of Oncology, Fudan University Shanghai Medical College, Shanghai, China; ^3^ Department of Breast Disease, The First Hospital of Jiaxing and The Affiliated Hospital of Jiaxing University, Jiaxing, China; ^4^ Department of Breast Surgery, The Third Affiliated Hospital of Kunming Medical University, Yunnan Cancer Hospital, Kunming, China; ^5^ Collaborative Innovation Center for Cancer Medicine, Shanghai Medical College, Fudan University, Shanghai, China

**Keywords:** breast cancer, CRISPR/Cas9, CCDC102B, RACK1, chaperone-mediated autophagy, NF-κB pathway

## Abstract

**Background:**

Breast cancer is one of the leading causes of cancer-related death among women, and the pathological status of axillary lymph nodes is an important predictor of prognosis. However, the mechanism involved in this early stage of metastasis remains largely unknown.

**Methods:**

Microarray analysis was used to carry out differential genomics analyses between matched pairs of metastatic sentinel lymph node tissues and breast primary tumors. The CRISPR/Cas9 gene editing system was used for *in vivo* screening by transplanting a loss-of-function cell pool into immunocompromised mice. MAGeCK was used to analyze the screening results. Survival analysis was performed *via* the Kaplan–Meier method. Cell proliferation, wound healing, migration and invasion assays were performed to confirm the phenotype. A tail vein model and subcutaneous xenotransplanted tumor model were used for the *in vivo* study. The relationship between coiled-coil domain containing 102B (CCDC102B) and receptor for activated C kinase 1 (RACK1) was examined using coimmunoprecipitation, mass spectrometry, nuclear protein extraction and immunofluorescence assays. The primary biological functions and pathways related to CCDC102B were enriched by RNA sequencing.

**Results:**

We identified CCDC102B through screening and found that it was significantly upregulated in metastatic lesions in lymph nodes compared to matched primary tumors. Increased expression of CCDC102B promoted breast cancer metastasis *in vitro* and *in vivo*. Additionally, high expression of CCDC102B was correlated with poor clinical outcomes in breast cancer patients. We further identified that CCDC102B was stabilized by the loss of RACK1, a protein negatively correlated with breast cancer metastasis. Mechanistically, we found that RACK1 promoted CCDC102B lysosomal degradation by mediating chaperone-mediated autophagy (CMA). The aggressive behavior of CCDC102B in breast cancer cells could be reversed by the expression of RACK1. Moreover, CCDC102B was correlated with the significant enrichment of NF-κB pathway components. Overexpressing CCDC102B led to less interaction between RACK1 and IKKa. Thus, CCDC102B positively regulates the NF−κB pathway by interacting with RACK1.

**Conclusion:**

Taken together, our findings uncover a novel role of CCDC102B in breast cancer metastasis. CCDC102B serves as a potential metastasis promoter by regulating the activation of the NF-κB pathway and can be degraded by RACK1 *via* CMA.

## Introduction

Breast cancer is one of the most common malignant diseases and the leading global cause of cancer-related deaths among women (1–3). With the progress of precision medicine, the overall survival of breast cancer patients has improved in recent years, but metastasis is still the leading cause of treatment failure and mortality (4, 5). Thus, it is important to understand the mechanisms underlying the metastatic process and find effective therapeutic targets. For breast cancer patients, the pathological status of axillary lymph nodes (LNs) is one of the most important predictors of prognosis (6, 7). Theoretically speaking, metastasis to the sentinel lymph nodes (SLNs) is the earliest stage of all kinds of metastasis. However, the mechanism involved in this early stage of metastasis remains largely unknown. Fortunately, the application of sentinel lymph node biopsy (SLNB) has provided us with an opportunity to study the mechanism of early-stage LN metastasis in breast cancer. Thus, novel metastasis-promoting genes may be identified by comparing the genetic changes between SLN metastasis tissues and primary tumor tissues.

The clustered regularly interspaced short palindromic repeats (CRISPR)-associated (Cas) protein 9 system, first discovered in bacteria as part of an adaptive immune system, is now a powerful genome editing technology that has been extensively applied in various cell types and organisms (8, 9). The CRISPR/Cas9 system includes a nonspecific Cas9 nuclease and a set of programmable sequence-specific CRISPR RNAs, which can guide Cas9 to cleave DNA and generate double-strand breaks at target sites (10, 11). Thus, the specificity of CRISPR/Cas9-mediated DNA cleavage requires target sequences matching CRISPR RNA. Recent advances in the CRISPR/Cas9 system enable the acceleration of cancer research by providing an efficient technology to dissect the mechanisms of tumorigenesis and identify targets for drug development, providing great promise for the treatment of cancer (8, 9, 12).

Coiled-coil domains, mostly composed of approximately 200 amino acids, are structural motifs that have been widely identified in proteins distributed in living creatures (13). Due to the specific coiled-coil structure, these proteins adopt different spatial confirmations to regulate a series of biological functions. Previous studies have indicated that coiled-coil domain containing (CCDC) proteins play important roles in tumorigenesis and progression through regulating intracellular signal transduction, the transcription of genetic information, the cell cycle, differentiation, apoptosis, etc. (14–18). Recently, increasing evidence has confirmed that CCDC proteins are associated with cancer metastasis (19–22). CCDC102B was first identified as an age-correlated DNA methylation marker for forensic use (23–25). With further research, CCDC102B was identified as a new centrosome linker protein that is required for maintaining centrosome cohesion and was found to be associated with the development of myopic maculopathy (26, 27). However, few studies have focused on the function and mechanism of CCDC102B in the development and metastasis of breast cancer, especially in the early stage of the transition from primary cancer to metastatic tumors.

The ubiquitin–proteasome system and autophagy–lysosome system are two main pathways for protein degradation in eukaryotes (28, 29). In general, the ubiquitin–proteasome system targets short-lived proteins for proteasome-mediated degradation through polyubiquitination (29). In contrast, the autophagy–lysosome system targets long-lived proteins and organelles for degradation in lysosomes (28). There are three types of autophagy involved in lysosomal degradation: macroautophagy, microautophagy, and chaperone-mediated autophagy (CMA) (28). Distinct from other autophagy types, the CMA pathway is the lysosomal degradation process in which substrates are selectively recognized by a cytosolic chaperone in the presence of a consensus pentapeptide called the Lys-Phe-Glu-Arg-Gln (KFERQ)-like motif (30). Substrates containing KFERQ-like motifs are recognized by HSPA8 and then bind to LAMP2A, a CMA receptor that mediates the translocation of substrate in the lysosomal lumen for degradation (28, 30–34).

In this study, we identified CCDC102B *via* CRISPR/Cas9 screening *in vivo* and found that it was significantly upregulated in metastatic LNs compared with primary tumors, which led to a poor prognosis in breast cancer patients. Overexpression of CCDC102B could promote breast cancer metastasis *in vitro* and *in vivo*. Mechanistically, receptor for activated C kinase 1 (RACK1), which was negatively correlated with breast cancer prognosis, bound to the third coiled-coil structure of CCDC102B, which includes two putative KFERQ-like motifs, and participated in the lysosomal degradation of CCDC102B through CMA. Furthermore, we found that CCDC102B could activate the NF−κB pathway by interacting with RACK1. Taken together, our findings demonstrate that CCDC102B plays a crucial role in promoting early-stage breast cancer metastasis and could serve as a novel predictor of clinical outcomes in breast cancer patients and a potential therapeutic target for mitigating breast cancer metastasis.

## Materials and Methods

### Cell Lines and Cell Culture

The MDA-MB-231, BT549 and HEK293T cell lines were purchased from the American Type Culture Collection (ATCC) (Manassas, VA, USA) and cultured according to ATCC instructions. The MDA-MB-231 BO cell line is a subline of MDA-MB-231 with a high osteolytic metastasis tendency and was a generous gift from Dr. Toshiyuki Yoneda (The University of Texas, USA) (35, 36). The MDA-MB-231 LM2 cell line is a subline of MDA-MB-231 cells with a high lung metastasis tendency, which was described previously (37). All cell lines were cultured in DMEM containing 10% FBS (Life Technologies), 100 U/ml penicillin and 100 μg/ml streptomycin (Invitrogen) at 37°C in a humidified incubator with 5% CO2. All cell lines were genotyped (Genewiz) and routinely tested for Mycoplasma contamination (Vazyme).

### Tissue Samples and Microarray Analysis

Five pairs of primary tumors and SLN macrometastatic loci were collected and confirmed by hematoxylin and eosin (H&E) staining at Fudan University Shanghai Cancer Center. Patients were preoperatively diagnosed with invasive ductal carcinoma (IDC) with clinically negative nodal status. The negative status of non-SLNs and a tumor cell percentage of over 70% in metastatic SLN loci were pathologically confirmed. All protocols were reviewed and approved by an independent institutional review board at Fudan University. All patients gave their written informed consent before inclusion in this study.

Primary tumor lesions and SLN macrometastatic loci were subjected to RNA extraction with TRIzol reagent. Paired samples were analyzed by Shanghai Qiming Biotechnology Co. Ltd. with Affymetrix GeneChip Human Transcriptome Array 2.0.

### RNA Isolation, RT–PCR and RT–qPCR

Total RNA was extracted from cells or tissues using TRIzol reagent (Thermo Fisher Scientific) according to the manufacturer’s protocol with DNase treatment. Subsequently, RNA was reverse transcribed into cDNA using oligo (dT) primers and a reverse transcription system according to the protocol of the RevertAid First Strand cDNA Synthesis Kit (Thermo Fisher Scientific). The single-stranded cDNA was amplified by PCR and overlap PCR using primers in **Supplementary Table 1**.

For RT–qPCR, RNA was reverse transcribed with the PrimeScript RT Reagent Kit (TaKaRa Biotechnology). Real-time PCR was performed with SYBR Premix Ex Taq (TaKaRa Biotechnology) using an ABI Prism 7900 instrument (Applied Biosystems). The relative expression of genes was quantified to ACTB (beta-actin) mRNA. Relative gene expression was calculated with the 2^−ΔΔCT^ method (38). The primer sequences used in this study are shown in **Supplementary Table 1**.

### CRISPR/Cas9 Knockout Pooled Library Cloning

DNA oligonucleotide library synthesis was performed on a microarray as previously described (39). The 1091 unique oligonucleotide sequences targeting 182 genes in our study were from the GeCKO library (**Supplementary Table 2**). Additionally, 409 nontargeted sequences were included as controls (**Supplementary Table 2**). A total of 1500 oligonucleotides with full lengths of 74 nt were amplified by PCR using Q5^®^ High-Fidelity DNA Polymerase (NEB) and purified on a 2% agarose E-Gel EX (Life Technologies) using the Wizard SV Gel and PCR Clean-up System (Promega). The lentiGuide-Puro vector (Addgene #52963) was digested with BsmBI (NEB) at 55°C for 2 hours and gel-purified on a 1% agarose E-Gel EX (Life Technologies) using a Wizard SV Gel and PCR Clean-up System (Promega). The Gibson ligation reaction (NEB) was performed using 10 ng of inserts and 25 ng of vector at 50°C for 30 min. From the ligation, 2 µl of the reaction was transformed into 50 µl of *E. coli* DH5α Electro-Cells (TaKaRa Biotechnology) according to the manufacturer’s protocol using a GenePulser (BioRad). To ensure representation, 8 parallel transformations were performed using the same ligation reaction and plated onto 245 mm×245 mm plates (Corning) with ampicillin (100 µg/ml) for selection, which yielded 300× library coverage with over 450,000 colonies. Plasmid DNA was extracted using the EndoFree Maxi Plasmid Kit (TIANGEN) after scraping the colonies off the plates.

### Lentivirus Packaging and Infection

The open reading frames (ORFs) of CCDC102B and RACK1 were all cloned from MDA-MB-231 cDNA and inserted into pCDH-CMV-Puro with or without a C-terminus 3×FLAG tag and pcDNA3.1-myc vector, respectively. The sgRNA oligos of CCDC102B and RACK1 were annealed and cloned into lentiGuide-Puro using BsmBI (**Supplementary Table 3**). Lentivirus was produced by cotransfecting HEK293T cells with specific plasmids together with psPAX2 and pMD2G. After 72 hours, viral supernatants were collected, filtered, and concentrated by ultracentrifugation. Lentivirus infected cells with the addition of polybrene (Sigma–Aldrich) at a working concentration of 8 μg/ml. Cells were incubated with lentivirus for 12 hours before changing the medium to FBS containing DMEM. For knockout, cells were transfected with lentiCas9-Blast (Addgene #52962) and lentiGuide-Puro. Infected cells were selected with puromycin (Sigma–Aldrich) at 2 μg/ml or blasticidin (Sigma–Aldrich) at 5 μg/ml for 1 week. For knockout cells, single colonies were found by seeding cells in 96-well plates at a very low density and confirmed by western blot.

### Cell Transduction by CRISPR/Cas9 Pooled Library

MDA-MB-231 BO-Cas9 cells were transduced with a CRISPR/Cas9 knockout pooled library *via* spinfection. To find the optimal volume of virus to achieve a multiplicity of infection (MOI) of 0.3, tests were performed by spinfecting 3.5×10^5^ cells with different volumes of virus. In detail, 3.5×10^5^ cells per well were plated into a 24-well plate with polybrene at a working concentration of 8 μg/ml, and each well received different amounts of virus; a control group without transduction was also included (**Supplementary Table 4**). The 24-well plate was centrifuged at 2000 g for 2 hours at 37°C. After spinning, the media was changed to standard media without polybrene. The cells were incubated for 24 hours and trypsinized using trypsin (Corning). The cells in each well were divided equally into four wells, and three of them underwent puromycin selection. As soon as the cells were all dead in the control wells without transduction, the cells were counted to calculate the efficiency of transduction, which was defined as the cell count in wells with puromycin selection divided by the cell count in wells with no puromycin multiplied by 100 (**Supplementary Table 4**). Finally, the volume of virus yielding an MOI closest to 0.3 (80 μl) was selected. Large-scale cell transduction was conducted in the same way with 80 μl of virus per well, and cells were pooled together into large flasks for puromycin selection and further research.

### Animal Studies

For CRISPR/Cas9 screening *in vivo*, 2×10^6^ MDA-MB-231 BO-Cas9 or MDA-MB-231 BO-Cas9 lentiGuide-CRISPR cells in 100 μl of sterile PBS were injected into BALB/c nude mice (6-8 weeks old) *via* the tail vein. Bioluminescence imaging was carried out by an IVIS-200 system (Xenogen) with intraperitoneal injection of 150 mg/kg D-Luciferin in each mouse 8 weeks after injection to monitor metastasis in the lungs. Lungs were fixed in polyformaldehyde for paraffin embedding.

To create a subcutaneous xenotransplanted tumor model for CCDC102B research, 2×10^6^ MDA-MB-231 LM2 pCDH-3×FLAG or MDA-MB-231 LM2 pCDH-3×FLAG-CCDC102B cells in 100 μl of sterile PBS were injected subcutaneously into fat pats of nude mice. Tumor progression was monitored by measuring the diameter of the tumor every 3 days. The xenografts were fixed in polyformaldehyde for paraffin embedding. To investigate tumor metastasis, 1×10^6^ MDA-MB-231 LM2 pCDH-3×FLAG or MDA-MB-231 LM2 pCDH-3×FLAG-CCDC102B cells were injected intravenously. Tumor metastasis to the lungs was monitored by bioluminescence imaging. The lungs were fixed in polyformaldehyde for paraffin embedding.

BALB/c nude mice were housed under specific-pathogen-free (SPF) conditions at the animal care facility of Shanghai Laboratory Animal Center (SLAC). All animal procedures were conducted in compliance with relevant institutional and national guidelines and regulations of Shanghai Medical Experimental Animal Care Commission.

### Deep Sequencing Processing

To confirm the plasmid DNA of the CRISPR/Cas9 knockout pooled library, deep sequencing was performed as described (39). Agencourt AMPure XP Bead Clean-Up (NEB) was used for DNA purification after each PCR step using High-Fidelity 2X PCR Master Mix (NEB). Amplicons from the second PCR were attached to Illumina adaptors and barcodes and sequenced using a HiSeq 3000 (Illumina). Primer sequences to amplify sgRNAs in each step are shown in **Supplementary Table 1**.

Genomic DNA from metastatic lungs and MDA-MB-231 BO-Cas9 lentiGuide-CRISPR cells before animal injection was harvested using the DNeasy Blood & Tissue Kit (Qiagen) according to the manufacturer’s protocol. The amount of genomic DNA of human transduced cells in mouse metastatic lungs was calculated as previously described (40). Two steps of PCR and DNA purification were performed as described above. To achieve 300× coverage over the library, the amount of input genomic DNA in each sample was calculated for the first PCR as described (39). Amplified sgRNA target sites were subjected to high-throughput genomic DNA sequencing using a HiSeq 3000 (Illumina). Primer sequences for the human-specific standard curve are shown in **Supplementary Table 1**.

All sequencing datasets were evaluated using FastQC (version 0.11.2) to ensure high quality. We mapped the reads to the reference genomic sequence and analyzed these datasets using MAGeCK analysis (41).

### RNA-Seq Analysis and Gene Set Enrichment Analysis (GSEA)

MDA-MB-231 cells were transduced with control or CCDC102B, and total RNA was extracted using TRIzol reagent. For RNA‐Seq analysis of cell lines (CCDC102B overexpressing versus control), the raw sequencing reads were aligned to the human reference genome (release version hg19) using TopHat. Gene expression levels were calculated according to the fragments per kilobase of transcript per million mapped reads (FPKM) values. GSEA was used to identify pathways enriched among differentially expressed genes.

### Study Population

Patients with invasive ductal breast cancer who were diagnosed and operated on between January 2008 and December 2009 in Fudan University Shanghai Cancer Center (FUSCC) with no neoadjuvant therapy were included. Paraffin-embedded tissues and clinical data for these patients were collected. A total of 212 patients were included with a median follow-up of 74.5 months. Diagnosis was verified by two independent pathologists in the Department of Pathology of FUSCC. Postoperative therapy was suggested according to guidelines. This study was approved by the Ethics Committee of FUSCC, and all participants signed informed consent documents.

### Tissue Microarrays (TMAs) and IHC Staining

TMAs were constructed from paraffin-embedded breast cancer samples obtained from the breast cancer patients described above. Tissue cylinders 2 mm in diameter were punched from marked tumor areas in each sample and inserted into a recipient paraffin wax block. Each sample was punched twice into the microarray to compare staining patterns in different areas of the same tumor in the same patient. Paraffin-embedded 4-mm-thick slides of TMAs were made and stained with 1:200 CCDC102B antibody (Abcam) using standard immunohistochemistry (IHC) protocols. Scores were calculated by multiplying the percentage of cells (scale: 0–3) and the intensity of protein expression (scale: 0–3). Procedures were conducted by experienced pathologists. IHC scores were verified by two independent researchers. All protocols were reviewed and approved by the review board at Fudan University. Informed consent was obtained from all patients. In addition, paraffin-embedded samples of xenotransplanted tumors and metastatic lungs were sliced and stained with 1:200 CCDC102B antibody (Abcam).

### Western Blot Analysis

Total cell lysates were extracted, and western blot analysis was conducted according to standard procedures. Briefly, protein lysates from cultured cells were harvested with RIPA buffer containing proteinase and phosphatase inhibitors. Samples were resolved by SDS–PAGE and transferred to PVDF membranes (Millipore). The membranes were blocked in 5% skim milk or 5% BSA and incubated with primary antibodies and then HRP-conjugated secondary antibodies. The antibodies used in this study are listed in **Supplementary Table 5**.

### Cell Proliferation, Wound Healing and Transwell Assays

Cell proliferation, wound healing and Transwell assays were performed as previously described (42). Cell proliferation and wound healing were imaged by an IncuCyte ZOOM System (Essen Bioscience). For the Transwell assay, cells were incubated at 37°C for different times according to their migration ability. The images of three random fields of migrated cells were analyzed by ImageJ. All experiments were conducted in triplicate.

### Coimmunoprecipitation (Co-IP) and Mass Spectrometry

Lentivirus-infected cells with a 3×FLAG tag were collected, and IP lysis buffer (Thermo) with protease inhibitor cocktail (Thermo) was added. Cell lysates were put on ice for 30 min followed by centrifugation at 12000 rpm for 15 min at 4°C. Meanwhile, magnetic beads (Sigma) were washed with PBS and blocked in 5% BSA for 1 hour at 4°C. The beads were washed again 3 times with IP lysis buffer. After spinning the cell lysates, a 5% volume of supernatant was saved as input, and the rest of the supernatant was added to beads for incubation overnight at 4°C. The next day, the beads were washed 6 times with IP lysis buffer. Proteins were eluted by 0.1 M glycine-hydrochloride solution and boiled with 1x SDS loading buffer as well as input. Samples were subjected to western blotting and resolved with SDS–PAGE. The protein gel was fixed with glacial acetic acid as a fixing solution and silver stained with a Fast Silver Stain Kit (Beyotime) according to the manufacturer’s protocol. Differential bands of different lentivirus-infected cells were cut and analyzed by LC–MS/MS (Shanghai Applied Protein Technology, Shanghai, China). MS spectra were searched using MASCOT version 2.4.01 (Matrix Science, London, UK) against the RefSeq human protein database (National Center for Biotechnology Information). For the IP experiment, western blotting was used with standard protocols to test the immunoprecipitated proteins.

### Immunofluorescence

MDA-MB-231 cell lines grown on coverslips were fixed with 4% paraformaldehyde for 30 min at room temperature, permeabilized with 0.2% Triton X-100 for 10 min at room temperature, blocked in 1% BSA for 1 hour at room temperature and incubated with primary antibodies overnight at 4°C. The slides were then incubated with Alexa 555–conjugated or Alexa 488–conjugated secondary antibodies (Cell Signaling Technology) for 2 hours at room temperature. DNA staining was performed using Fluoroshield Mounting Medium with DAPI (Abcam). Images were captured with a Leica SP5 confocal laser microscope (Leica Microsystems, Buffalo Grove, USA).

### Statistical Analysis

Statistical analyses were performed using SPSS version 23.0 and Prism GraphPad 8.0. T tests were used to calculate the P values for most of the experiments. Survival curves were plotted using the Kaplan–Meier method and were compared using log-rank tests. The experiments were repeated three times independently with similar results obtained, and the results of a representative experiment are shown in the tables or figures.

## Results

### CRISPR Screening *In Vivo* Identified CCDC102B as a Candidate Metastasis Promoter in Breast Cancer

**Figure 1:**
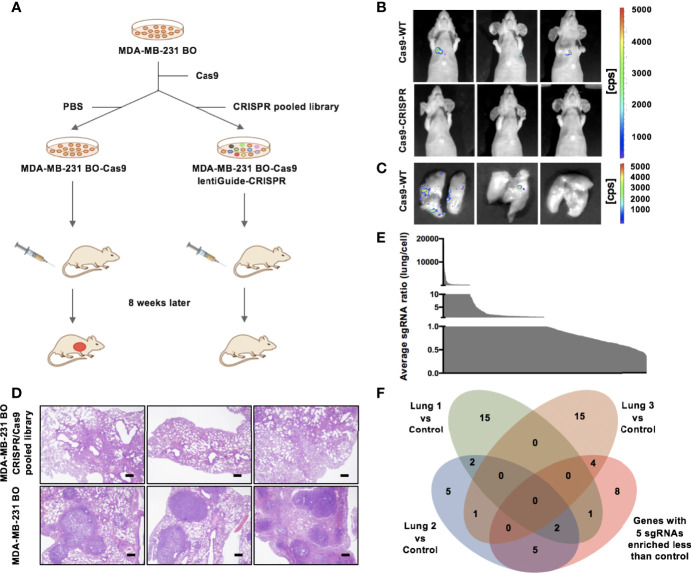
CRISPR/Cas9 screening identifies CCDC102B as a metastasis promoter. **(A)** Outline of the CRISPR/Cas9 screening strategy. **(B)** BLI of three representative mice in each group 8 weeks after injection with transduced cells. (n=6 of each group) **(C)** BLI of three representative lungs in control group mice with metastasis. (n=6 of each group) **(D)** Representative images of H&E staining of lung metastasis in each group 8 weeks after injection with transduced cells. Scale bars: 500 μm. (n=6 of each group) **(E)** Average ratio of all individual sgRNAs in lungs versus cell pool. **(F)** Venn diagram of candidate breast cancer metastasis promoter selection.

Gene microarray and comparative genomics analyses were used to define the differentially expressed genes between metastatic SLNs and matched primary tumors in breast cancer patients (**Supplementary Table 1A**, **Supplementary Figures 1A–C**). To identify candidate functional metastasis promoters in breast cancer, we chose genes that were 1.5-fold upregulated in metastatic SLNs compared with matched primary tumors and performed a CRISPR/Cas9-based knockout screen in MDA-MB-231 BO cells. We infected MDA-MB-231 BO cells with the CRISPR/Cas9 knockout pooled library of 2000 single-guide RNAs (sgRNAs) targeting 182 genes and injected 2×10^6^ transduced cells into the tail vein of immunocompromised BALB/c nude mice (**Figure 1A**). After 8 weeks, 75% (n=4) of mice that received the control cells had lung metastasis, whereas only one out of six mice that received the sgRNA library showed metastasis (**Figures 1B–D**).

To identify candidate sgRNAs that drive breast cancer metastasis, we used high-throughput sequencing and MAGeCK analysis to assess the representation of sgRNAs in three lungs that received the sgRNA library and the preinjected cells with the CRISPR pooled library (**Supplementary Table 1B**, **Supplementary Figures 1D–F**). The average ratio of all individual sgRNAs in the lungs versus the cell pool was measured (**Figure 1E**). Although the sgRNA library consisted of ~6 sgRNAs per gene, no experimental group showed greater enrichment of all 6 sgRNAs than the control group. We listed all 20 targets for which 5 sgRNAs were more enriched in the control group than the experimental groups (**Supplementary Table 1C**). Additionally, we listed the top 20 genes that were the least enriched in metastatic lungs compared to the initial cell pool according to MAGeCK analysis (**Supplementary Table 1D**). We merged the results of these lists (**Figure 1F** and **Supplementary Table 1E**), which revealed genes not only targeting known tumor promoters (e.g., JAK3 and SELL) and tumor promoters with demonstrated roles in other tissues (e.g., CD27, PROX1 and PSIP1) but also genes not previously described as tumor promoters (e.g., CCDC102B and C16orf54). These results showed that our approach was suited to identifying metastasis promoters in breast cancer. We identified CCDC102B, newly described as a tumor-related gene, as a candidate metastasis promoter in breast cancer.

### Increased Expression of CCDC102B Correlates With Poor Clinical Outcomes in Breast Cancer

**Figure 2:**
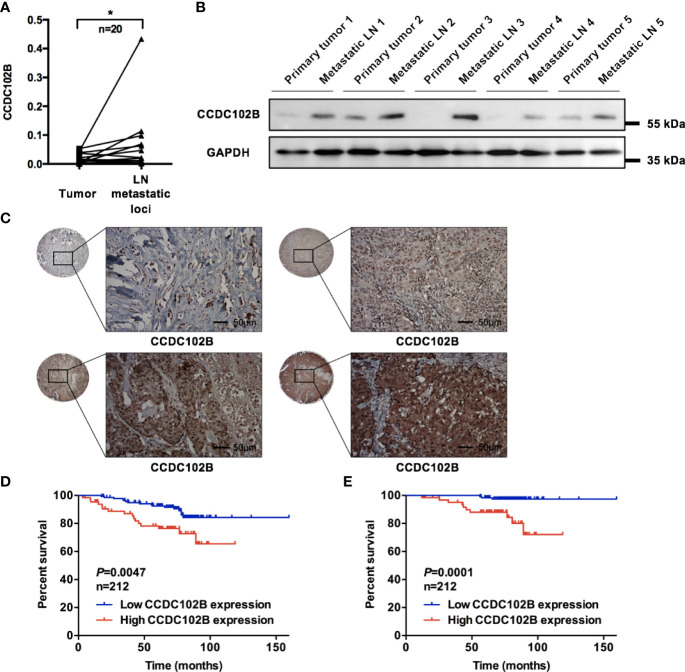
Increased expression of CCDC102B correlates with poor clinical outcome. **(A)** Relative expression of CCDC102B in primary breast cancer tissues (tumor) and their corresponding LN metastatic loci in a 20-paired-sample cohort (Wilcoxon matched-pairs signed rank test). Normalized to β-ACTIN. (*P=0.011) **(B)** Expression of CCDC102B in primary tumors and corresponding metastatic LNs. **(C)** Breast tumor tissue microarray from 212 breast cancer patients using immunohistochemistry to determine CCDC102B expression. Scale bars: 50 μm. **(D, E)** Kaplan-Meier analysis and Log-rank test of recurrence-free survival (RFS) and overall survival (OS) with different expression of CCDC102B in breast cancer patients. *P ≤ 0.05.

To validate the increase in CCDC102B in LN metastatic loci, we analyzed its expression by qRT–PCR in breast cancer patients with LN metastasis (n=20). CCDC102B expression was significantly increased in LN metastatic loci compared with primary tumors (*P*=0.011, **Figure 2A**). Further examination of the expression of CCDC102B by western blot analysis of primary tumors and corresponding metastatic LNs (n=5) showed that CCDC102B expression was significantly higher in metastatic LNs than in primary tumors (**Figure 2B**). To explore the role of CCDC102B in breast cancer, we examined the expression of CCDC102B in 212 breast tumor samples and analyzed the correlation between CCDC102B expression and the clinicopathological characteristics of breast cancer patients (**Supplementary Table 2A**). IHC staining of a TMA confirmed that CCDC102B was expressed in breast tumor tissues (**Figure 2C**), and semiquantitative scoring revealed that the high expression of CCDC102B was correlated with younger age (*P*=0.033) and higher histological grade (*P*=0.019). We found that high expression of CCDC102B was associated with LN metastasis (*P*=0.052) (**Supplementary Figure 2A**). In addition, the bc-GenExMiner 3.0 database was used to explore the correlation between CCDC102B expression and nodal status in breast cancer patients (43). With analysis of targeted expression of CCDC102B in breast cancer patients with different nodal status, we found significantly higher expression of CCDC102B in LN-positive patients (*P*=0.0005, **Supplementary Figure 2B**).

Based on the TMA data, we analyzed the correlation between CCDC102B expression and the prognosis of breast cancer patients, which showed that CCDC102B was an independent predictive factor of both recurrence-free survival (RFS) and overall survival (OS) (*P*=0.009 and *P*=0.002, respectively, **Supplementary Tables 2B, C**). Kaplan–Meier analysis indicated that high expression of CCDC102B was associated with poor clinical outcomes. When all patients were considered as a whole group, patients with high CCDC102B expression showed significantly reduced RFS and OS compared with that of those with low CCDC102B expression (*P*=0.0047 and *P*=0.0001, respectively, **Figures 2D, E**), which was similar to the clinical outcomes observed from the Gene Expression Omnibus (GEO) database (Affymetrix HGU133A and HGU133+2 microarrays) (44) (**Supplementary Figures 2C, D**). The median survival time of breast cancer patients with high CCDC102B expression was 70.30 months, which was significantly shorter than that of patients with low CCDC102B expression (75.87 months). When these tumors were divided into luminal A, luminal B, HER2+ and triple-negative breast cancer (TNBC) subtypes, high expression of CCDC102B showed a particularly strong correlation with poor clinical outcome in HER2+ and TNBC (**Supplementary Figures 2E, F**). Taken together, these results demonstrated that increased expression of CCDC102B was correlated with poor clinical outcomes in breast cancer, suggesting that CCDC102B was associated with increased metastatic ability in breast cancer.

### CCDC102B Promotes Breast Cancer Cell Migration and Metastasis *In Vitro* and *In Vivo*

**Figure 3:**
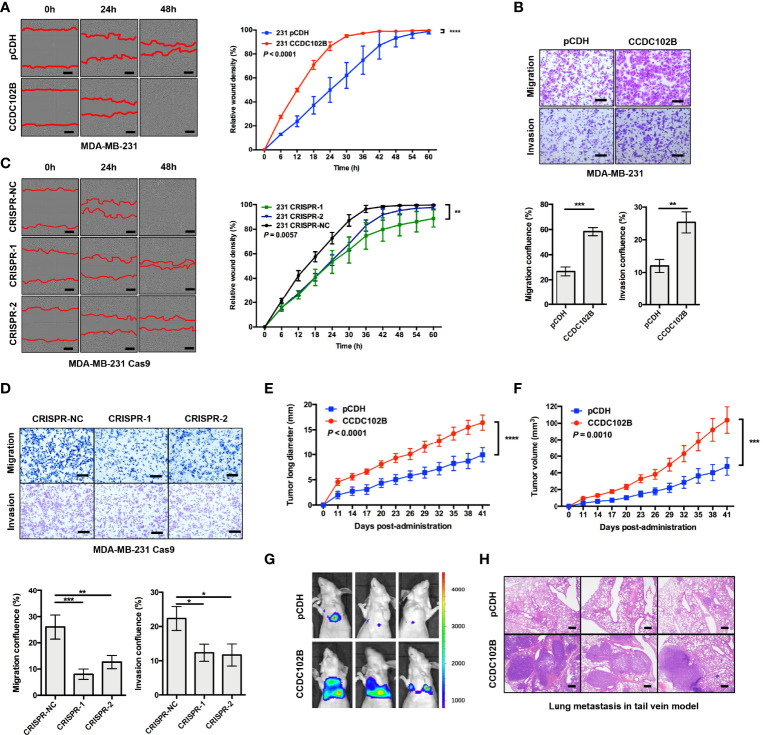
CCDC102B promotes breast cancer metastasis. **(A, B)** Wound healing assay and Transwell assay in MDA-MB-231 cells. CCDC102B overexpressed cells and the corresponding control cells were used. Representative photos and quantitative analysis were shown. Relative wound density was determined by IncuCyte. Confluence of Transwell was analyzed by ImageJ. n=3, biological replicates. Scale bars: 100 μm. **(C, D)** Wound healing assay and Transwell assay in MDA-MB-231 Cas9 cells. CCDC102B KO cells by CRISPR system and the corresponding control cells were used. Representative photos and quantitative analysis were shown. Relative wound density was determined by IncuCyte. Confluence of Transwell was analyzed by Image J. n=3, biological replicates. Scale bars: 100 μm. **(E, F)** Tumor long diameter and tumor volume were measured in orthotopic xenograft breast cancer model with overexpressed CCDC102B cells and the corresponding control cells. (Unpaired t test was performed on the day 41, n=12 of each group) **(G)** BLI of three representative mice in each group 6 weeks after injection with transduced cells in tail vein model. (n=6 of each group) **(H)** Representative images of H&E staining of lung metastasis in each group 6 weeks after injection with transduced cells in tail vein model. (n=6 of each group) Scale bars: 500 μm. *P ≤ 0.05; **P ≤ 0.01; ***P ≤ 0.001; ****P ≤ 0.0001.

To investigate the role of CCDC102B in breast cancer migration and metastasis, we analyzed the expression of CCDC102B in breast cancer cells (**Supplementary Figure 3A**) and developed three CCDC102B-overexpressing stable breast cancer cell line models (MDA-MB-231, BT549, and MDA-MB-231 LM2) for *in vitro* and *in vivo* studies. Western blot analysis and qRT–PCR both demonstrated that the CCDC102B expression level was strongly increased (**Supplementary Figure 3B**). In parallel, stable CCDC102B-targeting CRISPR/Cas9 knockout breast cancer cell line models (MDA-MB-231 and BT549) were established with distinct sgRNAs. Single colonies were selected from breast cancer cells transduced with CCDC102B-targeting CRISPR/Cas9, and knockout efficiency was examined with western blot analysis and qRT–PCR (**Supplementary Figure 3C**). Overexpression of CCDC102B significantly enhanced the migration and invasion ability in Transwell assays of MDA-MB-231, BT549, and MDA-MB-231 LM2 cells (**Figures 3A, B** and **Supplementary Figures 3D–F**) and also increased proliferation (**Supplementary Figure 3G**). In contrast, knockout of endogenous CCDC102B in MDA-MB-231 and BT549 cells resulted in significant decreases in proliferation, migration and invasion (**Figures 3C, D** and **Supplementary Figures 3H–J**).

To further validate the potential significance of CCDC102B in breast cancer metastasis *in vivo*, we generated an orthotopic xenograft breast cancer model using stable CCDC102B-overexpressing MDA-MB-231 LM2 cells. The growth of xenograft tumors and body weight were monitored. We found that the long tumor diameters and tumor volumes were significantly increased when CCDC102B was overexpressed (**Figures 3E, F** and **Supplementary Figure 3K**), while body weight was similar in the two groups (**Supplementary Figure 3L**). In addition, we developed a metastasis model with intravenous delivery of stable CCDC102B-overexpressing MDA-MB-231 LM2 cells with a retroviral construct expressing a GFP/luciferase fusion protein into the tail vein of nude mice. Tumor cell colonization and outgrowth in the lungs were monitored 6 weeks after the injection. We observed significantly increased lung metastasis in mice injected with CCDC102B-overexpressing cells compared with corresponding control mice with similar body weights in the two groups (**Figures 3G, H** and **Supplementary Figure 3M**). Taken together, these findings confirmed that high CCDC102B expression promoted breast cancer cell metastasis *in vitro* and *in vivo*.

### RACK1 Decreases the Stability of CCDC102B Through the Lysosome Pathway

**Figure 4:**
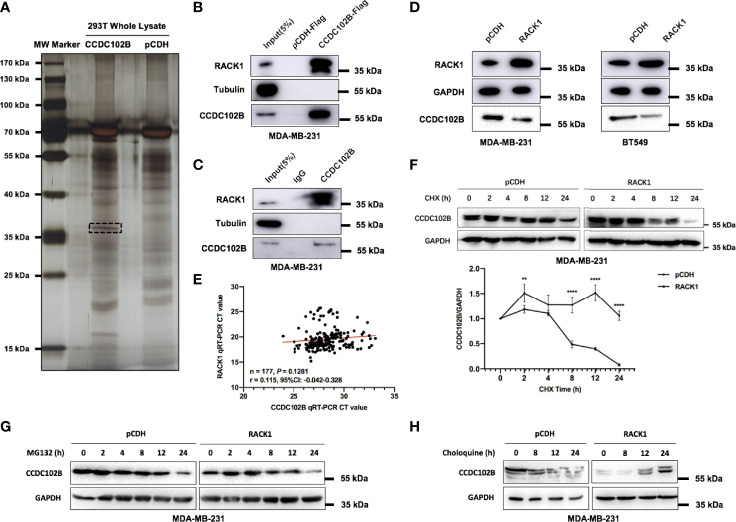
CCDC102B interacts with RACK1. **(A)** Co-IP identified potential interaction partners binding to CCDC102B. **(B)** Immunoprecipitation experiments validated interaction between CCDC102B and RACK1 using MDA-MB-231 stably overexpressing CCDC102B-Flag. **(C)** Immunoprecipitation experiments to validate endogenous interaction between CCDC102B and RACK1. **(D)** Overexpression of RACK1 significantly decreased the expression level of CCDC102B in both MDA-MB-231 cells and BT549 cells. **(E)** Gene expression profile correlation between CCDC102B and RACK1 using qRT-PCR analysis in 177 breast cancer patients. (Spearman r) **(F)** Overexpression RACK1 decreased the stability of CCDC102B and shortened the half-life of CCDC102B. Quantitative analysis was analyzed by ImageJ. (**P = 0.0010; ****P < 0.0001) n=3, biological replicates. **(G)** MG132 treatment did not significantly alter the protein levels of CCDC102B. **(H)** Choloquine treatment resulted in an accumulation of CCDC102B in a time-dependent manner.

To explore the underlying mechanism of CCDC102B-induced promotion of tumor metastasis, co-IP followed by mass spectrometry (MS) was employed to identify potential interaction partners binding to CCDC102B. RACK1 was identified as a major protein in CCDC102B-overexpressing samples (**Figure 4A** and **Supplementary Figure 4A**, **Supplementary Table 4A**). Next, we validated the interaction between CCDC102B and RACK1 by IP experiments using MDA-MB-231 cells stably overexpressing CCDC102B-Flag (**Figure 4B**). The endogenous interaction was further confirmed (**Figure 4C**).

RACK1, which contains seven Trp-Asp 40 (WD40) repeats, is a scaffold protein with diverse functions that has been reported to interact with several receptors and regulate signaling pathways (45–49). To determine the functional significance of the association between CCDC102B and RACK1, we examined the expression level of RACK1 (**Supplementary Figure 4B**). Interestingly, we noticed that overexpression of RACK1 significantly decreased the expression level of CCDC102B in MDA-MB-231 and BT549 cells (**Figure 4D**). In contrast, CRISPR/Cas9 knockout of RACK1 led to increased expression of CCDC102B (**Supplementary Figure 4C**). The negative correlation of RACK1 and CCDC102B expression was not due to repressed transcription, as revealed by qRT–PCR analysis in breast cancer patients (n=177) (**Figure 4E**). Similar results were shown with the bc-GenExMiner 3.0 database when analysis with gene correlation between CCDC102B and RACK1, indicating that there was little transcriptional correlation between CCDC102B and RACK1 (**Supplementary Figure 4D**) (43). Therefore, we hypothesized that the association between CCDC102B and RACK1 was mediated by cellular posttranslational processing. To investigate the effect of RACK1 on CCDC102B stability, MDA-MB-231 cells transfected with empty vector or RACK1 were incubated with 100 µM cycloheximide (CHX). The results showed that overexpression of RACK1 decreased the stability and shortened the half-life of CCDC102B (**Figure 4F**). Together, these results suggest that RACK1 promotes the degradation of CCDC102B.

The ubiquitin–proteasome system and autophagy–lysosome system are two main pathways for protein degradation in eukaryotes (28, 29). To delineate which pathway is responsible for RACK1-mediated CCDC102B degradation, MDA-MB-231 cells transfected with empty vector or RACK1 were treated with 2 µM proteasome inhibitor MG132 or 10 µM lysosome inhibitor chloroquine. We found that MG132 treatment did not significantly alter the protein levels of CCDC102B (**Figure 4G**), while chloroquine treatment resulted in the accumulation of CCDC102B in a time-dependent manner (**Figure 4H**). Thus, these data suggest that RACK1 promotes CCDC102B degradation mainly through the lysosome pathway.

### RACK1 Promotes CCDC102B Lysosomal Degradation by Mediating CMA

**Figure 5:**
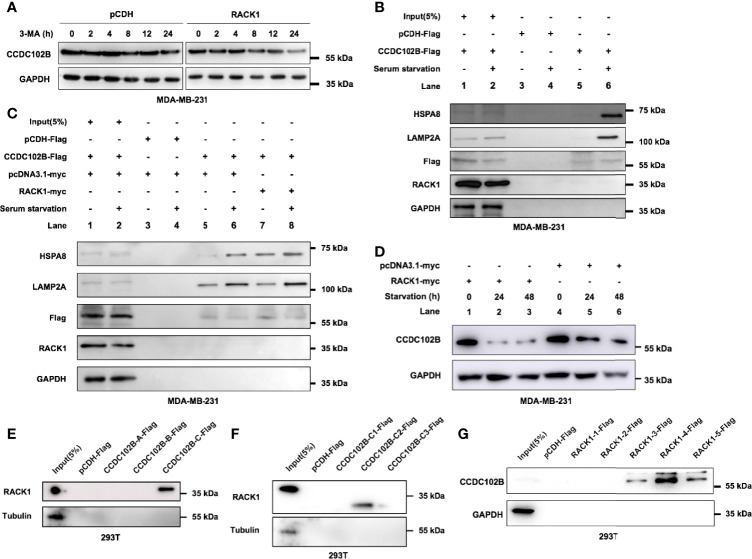
RACK1 promotes CCDC102B degradation. **(A)** 3-MA had no significant effects on the stability level of CCDC102B. **(B)** MDA-MB-231 cells were cultured in complete or serum-free medium for 24h, and then subjected to IP and immunoblotting. CCDC102B overexpressed cells and the corresponding control cells were used. **(C)** MDA-MB-231 cells were cultured in complete or serum-free medium for 24h, and then subjected to IP and immunoblotting. CCDC102B overexpressed cells, RACK1 overexpressed cells and the corresponding control cells were used. **(D)** MDA-MB-231 cells were cultured in serum-free medium for the indicated times and analyzed by immunoblotting. RACK1 overexpressed cells and the corresponding control cells were used. **(E)** RACK1 interacted with CCDC102B-C. (**F)** RACK1 interacted with CCDC102B-C2. **(G)** CCDC102B interact with RACK1-3.

There are three types of autophagy involved in lysosomal degradation. To determine whether macroautophagy contributes to the regulation of CCDC102B, 3-methyladenine (3-MA), a selective inhibitor of macroautophagy that blocks the formation of autophagosomes, was used to treat MDA-MB-231 cells transfected with empty vector or RACK1 vector. Western blot analysis showed that 5 mM 3-MA had no significant effects on the stability of CCDC102B (**Figure 5A**), which indicated that macroautophagy is not responsible for the lysosomal degradation of CCDC102B.

CMA is one of the three types of autophagy and is responsible for the selective degradation of proteins bearing a KFERQ-like motif (30). Interestingly, after reviewing the amino acid sequence of CCDC102B, we found seven putative KFERQ-like motifs at its N-terminus, namely, ^146^QKEAL^150^, ^156^QDLKL^160^, ^352^LERLQ^356^, ^374^QGLER^378^, ^386^QVKEM^390^, ^449^NRVDQ^453^ and ^490^QRSLD^494^, indicating that CCDC102B may be a CMA substrate. The KFERQ-like motif in the substrate is first selectively recognized by HSPA8, which recruits proteins to the lysosome, and then the substrate binds to LAMP2A, a CMA receptor that mediates the translocation of substrate in the lysosomal lumen for degradation (28, 30–34). To explore whether CCDC102B interacts with HSPA8 and LAMP2A, MDA-MB-231 cells transfected with empty vector or CCDC102B were cultured under serum starvation conditions, a well-established inducer of CMA, and IP assays were performed. The results showed that, compared with complete medium growth conditions, serum starvation enhanced the interactions between CCDC102B and HSPA8 and CCDC102B and LAMP2A (**Figure 5B**). In addition, overexpression of RACK1 further enhanced these interactions (**Figure 5C**). To demonstrate the role of RACK1 and CMA in CCDC102B degradation, we investigated the effect of RACK1 overexpression and serum starvation on the stability level of CCDC102B. Western blot analysis showed that the expression level of CCDC102B in MDA-MB-231 cells was decreased in a time-dependent manner following serum starvation and was further decreased by RACK1 overexpression (**Figure 5D**). Therefore, RACK1 enhanced the CMA-mediated lysosomal degradation of CCDC102B.

To further map the specific binding regions in CCDC102B and RACK1, a series of truncations were constructed according to the structures of the proteins with confirmation by western blot analysis (**Supplementary Figures 5A–C**). IP assays in 293T cells were performed with different truncations. The results showed that CCDC102B-C2, which included two KFERQ-like motifs, ^374^QGLER^378^ and ^386^QVKEM^390^, and the #5 WD40 domain in RACK1 were required for their interaction (**Figures 5F–H**). These data suggest that the interaction of RACK1 with the CCDC102B domain, including the KFERQ-like motif, may help expose the KFERQ-like motif and enhance recognition to promote CMA. Thus, RACK1 promotes CCDC102B lysosomal degradation by mediating CMA.

### RACK1 Decreases the Stability of CCDC102B and Inhibits Breast Cancer Metastasis

**Figure 6:**
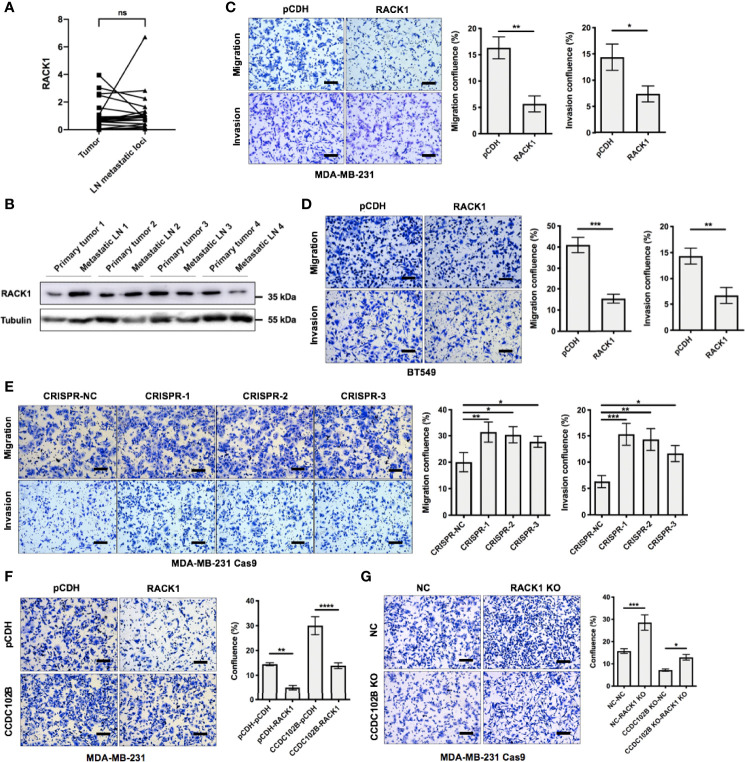
RACK1 inhibits breast cancer metastasis. **(A)** Relative expression of RACK1 in primary breast cancer tissues (tumor) and their corresponding LN metastatic loci in a 20-paired-sample cohort (Wilcoxon matched-pairs signed rank test). Normalized to β-ACTIN. **(B)** Expression of RACK1 in primary tumors and corresponding metastatic LNs. **(C, D)** Transwell assay in MDA-MB-231 cells and BT549 cells. RACK1 overexpressed cells and the corresponding control cells were used. Representative photos and quantitative analysis were shown. Confluence of Transwell was analyzed by ImageJ. n=3, biological replicates. Scale bars: 100 μm. **(E)** Transwell assay in MDA-MB-231 Cas9 cells. RACK1 KO cells by CRISPR system and the corresponding control cells were used. Representative photos and quantitative analysis were shown. Confluence of Transwell was analyzed by ImageJ. n=3, biological replicates. Scale bars: 100 μm. **(F, G)** Cell functional rescue tests with Transwell assay in MDA-MB-231 cells and MDA-MB-231 Cas9 cells. CCDC102B overexpressed cells, RACK1 overexpressed cells, CCDC102B KO cells, RACK1 KO cells and the corresponding control cells were used. Representative photos and quantitative analysis were shown. Confluence of Transwell was analyzed by ImageJ. n=3, biological replicates. Scale bars: 100 μm. ns, P > 0.05; *P ≤ 0.05; **P ≤ 0.01; ***P ≤ 0.001; ****P ≤ 0.0001.

Previous studies have shown that RACK1 regulates tumor metastasis by either promoting or inhibiting the progression of different tumor types and by regulating their microenvironments (47, 50–53). To validate the role of RACK1 in breast cancer, we analyzed the expression with qRT–PCR in breast cancer patients with LN metastasis (n=20) and found that RACK1 was similarly expressed in LN metastatic loci and primary tumors (*P*=0.7543, **Figure 6A**). Western blot analysis of primary tumors and corresponding metastatic LNs (n=4) also confirmed that RACK1 was similarly and ubiquitously expressed in both tissue types (**Figure 6B**). The bc-GenExMiner 3.0 database was used to explore the correlation between RACK1 expression and nodal status in breast cancer patients (43). With analysis of targeted expression of RACK1 in breast cancer patients with different nodal status, the results showed no significant difference in RACK1 expression between metastatic LN-positive and metastatic LN-negative patients (*P*=0.0936, **Supplementary Figure 6A**). In addition, clinical outcomes from the GEO database (Affymetrix HGU133A and HGU133+2 microarrays) (44) indicated that RACK1 expression was not associated with RFS in breast cancer patients (**Supplementary Figure 6B**), while OS was significantly correlated with RACK1 expression (**Supplementary Figure 6C**).

Furthermore, we investigated the role of RACK1 in breast cancer cell migration and metastasis. The results showed that overexpression of RACK1 significantly inhibited the migration and invasion of MDA-MB-231 and BT549 cells in Transwell assays (**Figures 6C, D**) and also slowed cell proliferation (**Supplementary Figure 6D**). However, there was no significant difference between the control and RACK1 overexpression groups in wound healing assays (**Supplementary Figure 6E**). In contrast, knockout of endogenous RACK1 in MDA-MB-231 cells resulted in the significant enhancement of migration and invasion (**Figure 6E**). These data suggest that RACK1 plays a role in metastasis inhibition in breast cancer.

Considering that RACK1 decreased the stability of CCDC102B, we hypothesized that RACK1 might exert its effects on breast cancer cell metastasis by regulating the degradation of CCDC102B. To verify this notion, functional rescue tests in breast cancer cells were performed. The results showed that the migration of MDA-MB-231 cells was significantly impaired by RACK1 overexpression, while RACK1-reduced cell migration was remarkably enhanced in CCDC102B-overexpressing cells (**Figure 6F**). On the other hand, the enhancement of the migration of RACK1 knockout breast cancer cells was attenuated by CCDC102B knockout (**Figure 6G**). Taken together, our results demonstrated that RACK1 inhibits breast cancer metastasis by decreasing the stability of CCDC102B.

### CCDC102B Activates the NF−κB Pathway by Interacting With RACK1

**Figure 7:**
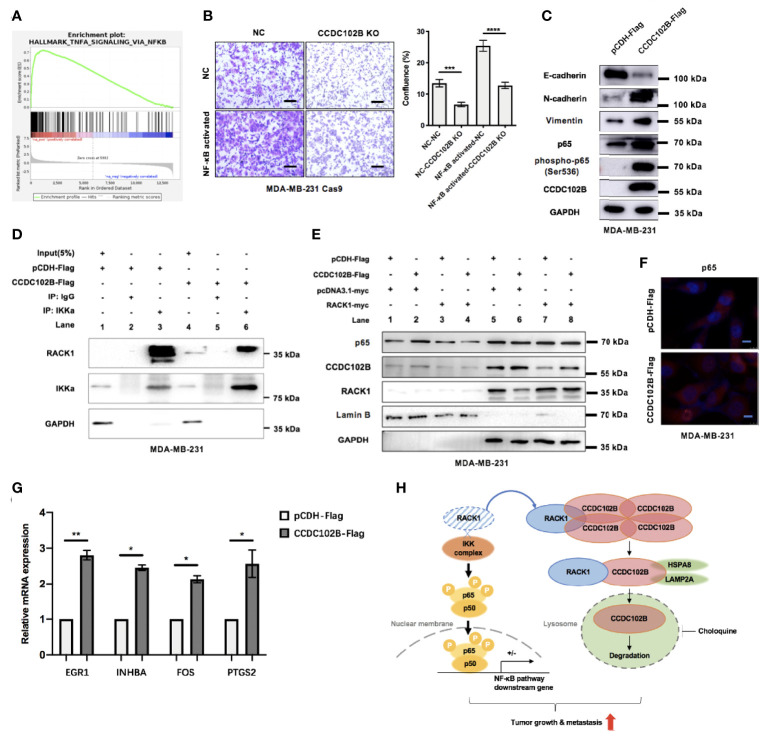
CCDC102B activates NF−κB pathway by interacting with RACK1. **(A)** GSEA showed CCDC102B was significantly associated with NF−κB pathway. **(B)** Cell functional rescue tests with Transwell assay in MDA-MB-231 Cas9 cells. CCDC102B KO cells, NF−κB activated cells and the corresponding control cells were used. Representative photos and quantitative analysis were shown. Confluence of Transwell was analyzed by ImageJ. n=3, biological replicates. Scale bars: 100 μm. **(C)** Overexpressed CCDC102B was associated with increased p65, phospho-p65 (Ser536) and EMT in MDA-MB-231. **(D)** Overexpressed CCDC102B led to less interaction between RACK1 and IKKα. **(E)** Movement of p65 into cell nucleus was promoted by CCDC102B, which could be decreased by overexpressed RACK1. **(F)** Immunofluorescence showed movement of p65 into cell nucleus promoted by CCDC102B. Scale bars: 10 μm. **(G)** Expression of NF−κB pathway target genes in CCDC102B-overexpressing MDA-MB-231 cells measured by qRT-PCR. n=3, biological replicates. **(H)** Schematic diagram of CCDC102B regulating breast cancer metastasis *via* NF−κB pathway. *P ≤ 0.05; **P ≤ 0.01; ***P ≤ 0.001; ****P ≤ 0.0001.

To further investigate the molecular mechanisms of increased metastasis in CCDC102B-overexpressing breast cancer cells, we performed RNA sequencing in MDA-MB-231 cells transfected with the CCDC102B-overexpressing vector and the corresponding control vector. Gene set enrichment analysis (GSEA) showed that genes with expression changes (fold change > 1.5) were significantly associated with cancer development and metastasis, such as the NF−κB pathway (**Figure 7A** and **Supplementary Table 7A, B**), supporting the role of CCDC102B in breast cancer cell proliferation and metastasis. Functional rescue tests were performed showing that the migration of breast cancer cells was significantly impaired by CCDC102B knockout, while was remarkably enhanced in NF−κB activated cells (**Figure 7B**). Western blot analysis confirmed that overexpressed CCDC102B was associated with increased p65, phospho-p65 (Ser536) and epithelial-mesenchymal transition (EMT) in MDA-MB-231 cells (**Figure 7C**). In addition, immunohistochemistry was performed in both orthotopic xenograft tumors and metastatic lungs in nude mice, showing that p65 was significantly increased in models with CCDC102B-overexpressing MDA-MB-231 LM2 cells (**Supplementary Figures 7A, B**).

Previous studies showed that RACK1, as a negative regulator of NF-κB signaling, interacts with the IKK complex and interferes with the recruitment of the IKK complex to TRAF2, which is a critical step for IKK phosphorylation (54). To further verify this, we performed functional rescue tests in breast cancer cells, showing that RACK1-reduced cell migration was remarkably enhanced in NF−κB activated cells (**Supplementary Figure 7C**). Western blot analysis also confirmed that RACK1 overexpression was associated with decreased p65 (**Supplementary Figure 7D**). However, IP assays showed that CCDC102B had no interaction with the IKK complex (**Supplementary Figure 7E**), suggesting that CCDC102B was not associated with the recruitment of the IKK complex. We hypothesized that CCDC102B activates the NF−κB pathway by interacting with RACK1, which decreases the interference with IKK complex recruitment and phosphorylation. To verify this hypothesis, we performed IP assays with MDA-MB-231 cells transfected with empty vector or CCDC102B vector. The results showed that, compared with the corresponding control cells, cells overexpressing CCDC102B exhibited less interaction between RACK1 and IKKa (**Figure 7D**). Moreover, we verified that the translocation of p65 into the cell nucleus was promoted by CCDC102B *via* western blot analysis and immunofluorescence (**Figures 7E, F**). Additionally, western blot analysis was performed to verify the decreased translocation of p65 into the cell nucleus with RACK1 overexpression (**Figure 7E**). We further analyzed the expression of several NF−κB pathway target genes showed in GSEA and confirmed that these highly ranked genes were overexpressed at transcription level in CCDC102B-overexpressing MDA-MB-231 cells (**Figure 7G**). Thus to sum up, CCDC102B positively regulates the NF−κB pathway by interacting with RACK1 (**Figure 7H**).

## Discussion

In this study, we presented several interesting findings concerning the biological function and regulatory mechanism of CCDC102B in breast cancer. For the first time, we identified CCDC102B as a significantly enriched protein in metastatic LNs compared to primary tumors *via* gene microarray analysis, comparative genomics analysis and *in vivo* CRISPR/Cas9-based knockout screening. We found that increased CCDC102B expression correlated with poor RFS and OS in breast cancer patients and could promote the migration and invasion of breast cancer cells. Moreover, overexpression of CCDC102B remarkably accelerated tumor growth and lung metastasis in breast cancer xenograft models. Therefore, CCDC102B could serve as an independent prognostic factor in breast cancer. Furthermore, we found a mutual effect between CCDC102B and RACK1. On the one hand, CCDC102B binds to RACK1 and interferes with the interaction between RACK1 and the IKK complex, which leads to activation of NF-κB signaling. On the other hand, RACK1 promotes the degradation of CCDC102B through CMA. Thus, the interaction between CCDC102B and RACK1 regulates the metastatic ability of breast cancer.

Previous studies have demonstrated the critical roles of RACK1 in regulating the processes of cancer cell proliferation, adhesion, migration, invasion and metastasis (45–49). RACK1, ubiquitously expressed in a wide range of tissues, was initially identified as an anchoring protein for protein kinase C (55). With an increasing number of reports showing its interaction with a large number of signaling transduction complexes and signaling pathways, such as Src family kinases, HIF-1, the Hedgehog pathway, the AKT pathway and the NF-κB pathway, RACK1 has been widely perceived as a platform for integrating diverse signaling activities (50–54, 56–59). The NF-κB pathway was also significantly enriched in CCDC102B-overexpressing breast cancer cells. Thus, all of these reports indicate the relationship among RACK1, CCDC102B and the NF-κB pathway, which has important functions in breast cancer metastasis. Generally, the NF-κB pathway comprises a family of transcription factors involved in the regulation of various biological response signaling pathways, which play a key role in cancer development, metastasis and resistance (60–63). Accordingly, NF-κB pathway activation increases NF-κB translocation in the nucleus and regulates gene expression. RACK1, identified as an IKK signalosome component, represses IKK activity and subsequent NF-κB activation through interaction with the IKK complex (54). In our study, we proposed that CCDC102B activates the NF−κB pathway by interacting with RACK1, which controls the sensitivity of NF-κB signaling by regulating IKK activation. Because CCDC102B was not found to be associated with the recruitment of the IKK complex, more questions about the mechanism of interaction among CCDC102B, RACK1 and the IKK complex were raised. One hypothesis is that CCDC102B competitively interacts with RACK1 with the same binding site of the IKK complex. Another hypothesis is that the interaction between CCDC102B and RACK1 influences the functional dimer formation of RACK1. Further research is needed to further clarify the mechanism.

Moreover, we provided evidence for the first time that CCDC102B undergoes lysosomal degradation *via* the CMA pathway. According to the mechanism by which cargo is delivered to lysosomes, autophagy can be classified as macroautophagy, microautophagy, or CMA (28). Distinct from other autophagy types, CMA is the first lysosomal degradation process in which substrates are selectively recognized by a cytosolic chaperone and tagged for degradation in lysosomes (32, 64). This process involves substrate recognition, unfolding, translocation, and degradation (32). By degrading specific target proteins, the CMA pathway is involved in a variety of cellular activities, including its critical role in the development of diseases (32, 64–68). CMA degrades proteins that contain a consensus pentapeptide called a KFERQ-like motif, which includes glutamine on one of the sides, one or two of the positive residues K and R, one or two of the hydrophobic residues F, L, I or V and one of the negatively charged E or D residues (30, 64). Proteins containing KFERQ-like motifs are recognized by HSPA8 and interact with LAMP2A, which helps with transportation to the lysosomal surface (28, 30–34). In this study, we demonstrated that CCDC102B, containing seven putative KFERQ-like motifs, was degraded in lysosomes by the CMA pathway. Serum starvation enhanced the interactions between CCDC102B and HSPA8 and between CCDC102B and LAMP2A. Furthermore, we found decreased expression of CCDC102B with serum starvation, which could be further decreased by RACK1 overexpression. Therefore, RACK1 enhanced the CMA-mediated lysosomal degradation of CCDC102B. With further mapping of specific binding regions in CCDC102B and RACK1, we found that RACK1, interacting with the CCDC102B domain including a KFERQ-like motif, promotes CCDC102B lysosomal degradation by mediating CMA. One possible mechanism of RACK1 was increased exposure of the KFERQ-like motif and enhanced recognition for the CMA process. Taken together, these results showed that CCDC102B protein levels are regulated by CMA-mediated lysosomal degradation, which could be enhanced by RACK1 overexpression.

Therefore, we believe that there is an interesting balance between CCDC102B and RACK1 (**Figure 7G**). When the expression level of CCDC102B is relatively low, which means that RACK1 is far more abundant than CCDC102B, the role of RACK1 is dominant; it promotes the CMA-mediated degradation of CCDC102B. Although CCDC102B can upregulate the NF-κB pathway, it is quickly degraded in the presence of excess RACK1. Generally, there will be no great impact on tumor development or metastasis. Nevertheless, when the expression level of CCDC102B is relatively high, which means RACK1 is far less abundant than CCDC102B, the role of CCDC102B is dominant. With little degradation, excess CCDC102B binds with RACK1, which may compete with IKK binding, resulting in NF-κB pathway activation and eventually promoting tumor development or metastasis. Thus, CCDC102B expression is one of the key predictors of early-stage metastasis in breast cancer.

In summary, we uncovered the interaction between CCDC102B and RACK1, which could be one of the mechanisms involved in the early-stage metastasis of breast cancer. As CCDC102B expression is upregulated in breast tumors, the discovery of novel drugs targeting CCDC102B could suppress breast cancer growth and mitigate metastasis. These emerging findings provide new mechanistic insights into early-stage breast cancer metastasis and define novel therapeutic targets for breast cancer.

## Data Availability Statement

The datasets presented in this study can be found in online repositories. The names of the repository/repositories and accession number(s) can be found in the article/**Supplementary Material**.

## Ethics Statement

The studies involving human participants were reviewed and approved by Fudan University. The patients/participants provided their written informed consent to participate in this study. The animal study was reviewed and approved by Shanghai Medical Experimental Animal Care Commission.

## Author Contributions

JS and YC designed and performed the experiments, analyzed data and wrote the paper. RG performed some of the experiments, analyzed data and provided some critical ideas. BX, WC, QZ, JC and JX performed some of the experiments and analyzed data. JC and JX provided the patients samples for clinical data analysis. JW and YC initiated the study, organized, designed, and revised the paper. All authors contributed to the article and approved the submitted version.

## Funding

This work was supported by grants from National Natural Science Foundation of China (Grant No. 81902674, 81874115, 82072919 and 81302297).

## Conflict of Interest

The authors declare that the research was conducted in the absence of any commercial or financial relationships that could be construed as a potential conflict of interest.

## Publisher’s Note

All claims expressed in this article are solely those of the authors and do not necessarily represent those of their affiliated organizations, or those of the publisher, the editors and the reviewers. Any product that may be evaluated in this article, or claim that may be made by its manufacturer, is not guaranteed or endorsed by the publisher.
