# Supplementary material for: Stabilization of CCDC102B by Loss of RACK1 Through the CMA Pathway Promotes Breast Cancer Metastasis via Activation of the NF-κB Pathway
Source: Front Oncol. 2022 Jul 25;12:927358. doi: 10.3389/fonc.2022.927358 (PMC9359432; doi:10.3389/fonc.2022.927358)
Supplement: Supplementary file 1 [file DataSheet_1.zip › supplementary/Supplementary Table 15 Positive signaling pathways of GSEA in CCDC102B overexpression MDA-MB-231.docx]

Supplementary Table 15 Positive signaling pathways of GSEA in CCDC102B overexpression MDA-MB-231

| PATHWAY | *P* value |
| --- | --- |
| TNFA_SIGNALING_VIA_NFKB | <0.001 |
| HYPOXIA | <0.001 |
| UNFOLDED_PROTEIN_RESPONSE | <0.001 |
| INFLAMMATORY_RESPONSE | <0.001 |
| HEDGEHOG_SIGNALING | <0.001 |
| P53_PATHWAY | <0.001 |
| KRAS_SIGNALING_UP | <0.001 |
| IL2_STAT5_SIGNALING | 0.001 |
| IL6_JAK_STAT3_SIGNALING | 0.007 |
| EPITHELIAL_MESENCHYMAL_TRANSITION | 0.01 |
| APOPTOSIS | 0.021 |
| UV_RESPONSE_UP | 0.03 |
| ESTROGEN_RESPONSE_EARLY | 0.03 |
