# Supplementary material for: Stabilization of CCDC102B by Loss of RACK1 Through the CMA Pathway Promotes Breast Cancer Metastasis via Activation of the NF-κB Pathway
Source: Front Oncol. 2022 Jul 25;12:927358. doi: 10.3389/fonc.2022.927358 (PMC9359432; doi:10.3389/fonc.2022.927358)
Supplement: Supplementary file 1 [file DataSheet_1.zip › supplementary/Supplementary Table 5 Antibodies used in the study.docx]

Supplementary Table 5 Antibodies used in the study

Anti-CCDC102B antibody (ab97810, Abcam)

Anti-RACK1 antibody (ab129084, Abcam)

Anti-HSPA8 antibody (ab51052, Abcam)

Anti-LAMP2A antibody (ab125068, Abcam)

Anti-DDDDK tag antibody (ab205606, Abcam)

Anti-E-Cadherin (4A2, #14472, Cell Signaling Technology)

Anti-N-Cadherin (D4R1H, #13116, Cell Signaling Technology)

Anti-Vimentin (D21H3, #5741, Cell Signaling Technology)

Anti-NF-κB p65 (D14E12, #8242, Cell Signaling Technology)

Anti-Phospho-NF-κB p65 (Ser536) (93H1, #3033, Cell Signaling Technology)

Anti-IKKα antibody (3G12, #11930, Cell Signaling Technology)

Anti-HRP-Conjugated GAPDH Antibody (HRP-60004, 1E6D9, Proteintech)

Anti-Lamin B1 antibody (ab133741, Abcam)

Anti-α-Tubulin (DM1A, #3873, Cell Signaling Technology)

Anti-rabbit IgG, HRP-linked Antibody (#7074, Cell Signaling Technology)

Anti-mouse IgG, HRP-linked Antibody (#7076, Cell Signaling Technology)
