# Supplementary material for: Stabilization of CCDC102B by Loss of RACK1 Through the CMA Pathway Promotes Breast Cancer Metastasis via Activation of the NF-κB Pathway
Source: Front Oncol. 2022 Jul 25;12:927358. doi: 10.3389/fonc.2022.927358 (PMC9359432; doi:10.3389/fonc.2022.927358)
Supplement: Supplementary file 1 [file DataSheet_1.zip › supplementary/Supplementary Table 11 Clinicopathological characteristics of breast cancer patients in tissue microarray and correlation with CCDC102B expression.docx]

Supplementary Table 11 Clinicopathological characteristics of breast cancer patients in tissue microarray and correlation with CCDC102B expression

| Variables | Patients (n=212) | Low expression (%)  (n=147) | High expression (%)  (n=65) | Univariate analysis  *P* value |
| --- | --- | --- | --- | --- |
| Age |  |  |  | 0.033 |
| ≤50 | 91 | 56 (38.1) | 35 (53.8) |  |
| >50 | 121 | 91 (61.9) | 30 (46.2) |  |
| Tumor size (pT) |  |  |  | 0.198 |
| ≤2cm (pT1) | 59 | 45 (30.6) | 14 (21.5) |  |
| 2-5cm (pT2) | 111 | 77 (52.4) | 34 (52.3) |  |
| >5cm (pT3) | 42 | 25 (17.0) | 17 (26.2) |  |
| LN status (pN) |  |  |  | 0.052 |
| Negative (pN0) | 119 | 89 (60.5) | 30 (46.2) |  |
| Positive (pN1-3) | 93 | 58 (39.5) | 35 (53.8) |  |
| Grade |  |  |  | 0.019 |
| I | 32 | 27 (18.4) | 5 (7.6) |  |
| II | 108 | 78 (53.0) | 30 (46.2) |  |
| III | 72 | 42 (28.6) | 30 (46.2) |  |
| LVI |  |  |  | 0.666 |
| Positive | 77 | 52 (35.4) | 25 (38.5) |  |
| Negative | 135 | 95 (64.6) | 40 (61.5) |  |
| ER |  |  |  | 0.590 |
| Positive | 107 | 76 (51.7) | 31 (47.7) |  |
| Negative | 105 | 71 (48.3) | 34 (52.3) |  |
| PR |  |  |  | 0.304 |
| Positive | 96 | 70 (47.6) | 26 (40.0) |  |
| Negative | 116 | 77 (52.4) | 39 (60.0) |  |
| HER2 |  |  |  | 0.777 |
| Positive | 78 | 55 (37.4) | 23 (35.4) |  |
| Negative | 134 | 92 (62.6) | 42 (64.6) |  |
| Ki67 |  |  |  | 0.504 |
| ≤14% | 147 | 104 (70.7) | 43 (66.2) |  |
| >14% | 65 | 43 (29.3) | 22 (33.8) |  |

Abbreviations: LN, lymph node; LVI, lymphovascular invasion; ER, estrogen receptor; PR, progesterone receptor; HER2, human epidermal growth factor receptor 2
