# Supplementary material for: Stabilization of CCDC102B by Loss of RACK1 Through the CMA Pathway Promotes Breast Cancer Metastasis via Activation of the NF-κB Pathway
Source: Front Oncol. 2022 Jul 25;12:927358. doi: 10.3389/fonc.2022.927358 (PMC9359432; doi:10.3389/fonc.2022.927358)
Supplement: Supplementary file 1 [file DataSheet_1.zip › supplementary/Supplementary Table 6 Patient characteristics of gene microarray.docx]

Supplementary Table 6 Patient characteristics of gene microarray.

| Patient | Tumor size (mm) | Grade | ER | PR | HER2 | Ki67 | SLN (+) | non-SLN (+) |
| --- | --- | --- | --- | --- | --- | --- | --- | --- |
| A | 17 | II | 100% | 90% | IHC- | 20% | 1 | 0 |
| B | 20 | II | 90% | 90% | IHC- | 40% | 2 | 0 |
| C | 22 | III | 70% | 70% | IHC- | 30% | 1 | 0 |
| D | 18 | II | 90% | 5% | FISH- | 20% | 1 | 0 |
| E | 20 | II | 90% | 90% | IHC- | 40% | 2 | 0 |

Abbreviations: ER, estrogen receptor; PR, progesterone receptor; HER2, human epidermal growth factor receptor 2; SLN, sentinel lymph node; IHC, immunohistochemistry; FISH: fluorescence in situ hybridization.
