# Supplementary material for: Stabilization of CCDC102B by Loss of RACK1 Through the CMA Pathway Promotes Breast Cancer Metastasis via Activation of the NF-κB Pathway
Source: Front Oncol. 2022 Jul 25;12:927358. doi: 10.3389/fonc.2022.927358 (PMC9359432; doi:10.3389/fonc.2022.927358)
Supplement: Supplementary file 1 [file DataSheet_1.zip › supplementary/Supplementary Table 8 The 20 targets for which 5 sgRNAs were enriched less than control.docx]

Supplementary Table 8 The 20 targets for which 5 sgRNAs were enriched less than control

| PTGDS | TMEM154 | SELL | BTLA | IL2RG |
| --- | --- | --- | --- | --- |
| CD5L | SP4 | WDFY4 | GCSAM | CCR7 |
| CD27 | FCRL1 | F8 | PSIP1 | EAF2 |
| PROX1 | ST6GAL1 | CR1 | CCDC102B | STAB2 |
