# Supplementary material for: Stabilization of CCDC102B by Loss of RACK1 Through the CMA Pathway Promotes Breast Cancer Metastasis via Activation of the NF-κB Pathway
Source: Front Oncol. 2022 Jul 25;12:927358. doi: 10.3389/fonc.2022.927358 (PMC9359432; doi:10.3389/fonc.2022.927358)
Supplement: Supplementary file 1 [file DataSheet_1.zip › supplementary/Supplementary Table 4 Spinfection test with different volumes of virus in order to achieve a MOI of 0.docx]

Supplementary Table 4 Spinfection test with different volumes of virus in order to achieve a MOI of 0.3.

| 7×10^5^/ml MDA-MB-231 BO-Cas9 (μl) | Virus (μl) | DMEM with 8μg polybrene (μl) | Percentage of transduction (%) |
| --- | --- | --- | --- |
| 500 | 0 | 500 | 0.0 |
| 500 | 5 | 495 | 1.6 |
| 500 | 10 | 490 | 5.5 |
| 500 | 20 | 480 | 7.9 |
| 500 | 40 | 460 | 16.9 |
| 500 | 80 | 420 | 33.3 |
| 500 | 120 | 380 | 80.8 |
| 500 | 160 | 340 | 98.2 |
| 500 | 200 | 300 | 93.6 |
| 500 | 250 | 250 | 115.9 |
| 500 | 300 | 200 | 95.7 |
| 500 | 400 | 100 | 70.0 |
