# Supplementary material for: Stabilization of CCDC102B by Loss of RACK1 Through the CMA Pathway Promotes Breast Cancer Metastasis via Activation of the NF-κB Pathway
Source: Front Oncol. 2022 Jul 25;12:927358. doi: 10.3389/fonc.2022.927358 (PMC9359432; doi:10.3389/fonc.2022.927358)
Supplement: Supplementary file 1 [file DataSheet_1.zip › supplementary/Supplementary Table 16 Negative signaling pathways of GSEA in CCDC102B overexpression MDA-MB-231.docx]

Supplementary Table 16 Negative signaling pathways of GSEA in CCDC102B overexpression MDA-MB-231

| PATHWAY | *P* value |
| --- | --- |
| E2F_TARGETS | <0.001 |
| G2M_CHECKPOINT | <0.001 |
| MITOTIC_SPINDLE | <0.001 |
| MYC_TARGETS_V1 | 0.014 |
| FATTY_ACID_METABOLISM | 0.016 |
| SPERMATOGENESIS | 0.02 |
| PEROXISOME | 0.038 |
