# Supplementary material for: Stabilization of CCDC102B by Loss of RACK1 Through the CMA Pathway Promotes Breast Cancer Metastasis via Activation of the NF-κB Pathway
Source: Front Oncol. 2022 Jul 25;12:927358. doi: 10.3389/fonc.2022.927358 (PMC9359432; doi:10.3389/fonc.2022.927358)
Supplement: Supplementary file 1 [file DataSheet_1.zip › supplementary/Supplementary Table 10 Merged results of Supplementary Table 8 and Supplementary Table 9.docx]

Supplementary Table 10 Merged results of Supplementary Table 8 and Supplementary Table 9

| Gene | Frequency of appearance in former lists |
| --- | --- |
| CCDC102B, CD5L | 3 |
| C16orf54, CD27, CORO1A, GCSAM, IL2RG, JAK3, PROX1, PSIP1, SELL, SP4, ST6GAL1, STAB2, WDFY4 | 2 |
| ABCD2, ATF7IP2, BACH2, BLNK, BTLA, CCR7, CD19, CD28, CD6, CD79A, CD79B, CETP, CLEC4M, CR1, CYTH1, EAF2, F8, FAM65B, FCRL1, FCRL2, FDCSP, FLT3, GIMAP1-GIMAP5, ITGA8, ITK, KCNA3, LOC256021, LRRN3, MARCO, MMRN1, P2RY10, PLA1A, PLCG2, PPM1K, PTGDS, RHOF, SLAMF1, SLAMF6, SMAP2, SP140, SPOCK2, STAP1, TMC8, TMEM154, TMEM71, TREML2, TSPAN7, UBASH3A | 1 |
