# Supplementary material for: Stabilization of CCDC102B by Loss of RACK1 Through the CMA Pathway Promotes Breast Cancer Metastasis via Activation of the NF-κB Pathway
Source: Front Oncol. 2022 Jul 25;12:927358. doi: 10.3389/fonc.2022.927358 (PMC9359432; doi:10.3389/fonc.2022.927358)
Supplement: Supplementary file 1 [file DataSheet_1.zip › supplementary/Supplementary file - 0416-UPDATE.docx]

**Supplementary file**

**Figure S1** CRISPR/Cas9 screening identifies metastasis promoters. **A.** The flow chart for screening candidate functional metastasis promoters in breast cancer. **B.** IHC of metastatic SLNs and matched primary tumors in breast cancer patients. Scale bars: black, 500 μm; pink, 100 μm. **C.** Heatmap of the differentially expressed genes between metastatic SLNs and matched primary tumors in breast cancer patients. **D., E. and F.** Mapping (**D**), missed sgRNAs (**E**) and intergroup correlation (**F**) in NGS results from three representative lungs that received the sgRNA library and the original CRIPSR pooled library.

**Figure S2** Correlation of CCDC102B expression with clinical outcome. **A.** CCDC102B expression in different LN status patients. **B.** Box and whisker plot of CCDC102B expression according to nodal status from bc-GenExMiner 3.0 database. **C.** and **D.** Kaplan-Meier analysis of recurrence-free survival (RFS) and overall survival (OS) with different expression of CCDC102B in breast cancer from GEO database. **E.** and **F.** Kaplan-Meier analysis and Log-rank test of recurrence-free survival (RFS) and overall survival (OS) in different subtypes of breast cancer patients.

**Figure S3** CCDC102B function in vitro and in vivo. **A.** Expression of CCDC102B in breast cancer cell lines measured by western blot analysis and qRT-PCR. n=3, biological replicates. **B.** CCDC102B was overexpressed in MDA-MB-231, BT549 and MDA-MB-231 LM2 cells measured by western blot analysis and qRT-PCR. n=3, biological replicates. **C.** CCDC102B was knocked out by CRIPSR/Cas9 in MDA-MB-231 Cas9 and BT549 Cas9 cells measured by western blot analysis and qRT-PCR. n=3, biological replicates. **D.** Wound healing assay in BT549 and MDA-MB-231 LM2 cells. CCDC102B overexpressed cells and the corresponding control cells were used. Quantitative analysis was shown. Relative wound density was determined by IncuCyte. n=3, biological replicates. **E.** and **F.** Transwell assay in BT549 and MDA-MB-231 LM2 cells. CCDC102B overexpressed cells and the corresponding control cells were used. Representative photos and quantitative analysis were shown. Confluence of Transwell was analyzed by ImageJ. n=3, biological replicates. Scale bars: 100 μm. **G.** Proliferation assay in MDA-MB-231, BT549 and MDA-MB-231 LM2 cells. CCDC102B overexpressed cells and the corresponding control cells were used. Quantitative analysis was shown. Confluence was analyzed by ImageJ. n=3, biological replicates. **H.** and **I.** Wound healing assay and Transwell assay in BT549 Cas9 cells. CCDC102B KO cells by CRISPR system and the corresponding control cells were used. Representative photos and quantitative analysis were shown. Relative wound density was determined by IncuCyte. Confluence of Transwell was analyzed by ImageJ. n=3, biological replicates. Scale bars: 100 μm. **J.** Proliferation assay in MDA-MB-231 Cas9 and BT549 Cas9 cells. CCDC102B KO cells by CRISPR system and the corresponding control cells were used. Quantitative analysis was shown. Confluence was analyzed by ImageJ. n=3, biological replicates. **K.** Tumor burden of orthotopic xenograft breast cancer model with overexpressed CCDC102B cells and the corresponding control cells. (n=12 of each group, one mouse in control group died due to non-tumor cause) **L.** Representative images of lung metastasis in each group 6 weeks after injection with transduced cells in tail vein model. (n=6 of each group) **M.** Weight of orthotopic xenograft breast cancer model mice in each group. (n=12 of each group) **N.** Weight of tail vein model mice in each group. (n=6 of each group)

**Figure S4** Interaction between CCDC102B and RACK1. **A.** Mass spectrometry (MS) assay result. **B.** Expression of RACK1 in breast cancer cell lines measured by western blot analysis and qRT-PCR. n=3, biological replicates. **C.** Knockout of RACK1 led to increase expression level of CCDC102B. **D.** Little transcriptional correlation between CCDC102B and RACK1. (Spearman r)

**Figure S5** Truncations in CCDC102B and RACK1. **A.** Truncations of CCDC102B-A, CCDC102B-B and CCDC102B-C. **B.** Truncations of CCDC102B-C1, CCDC102B-C2 and CCDC102B-C3. **C.** Truncations of RACK1-1, RACK1-2, RACK1-3, RACK1-4 and RACK1-5.

**Figure S6** Clinical outcome and cell function with overexpressed RACK1. **A.** Box and whisker plot of RACK1 expression according to nodal status from bc-GenExMiner 3.0 database. **B.** and **C.** Kaplan-Meier analysis of recurrence-free survival (RFS) and overall survival (OS) with different expression of RACK1 in breast cancer from GEO database. **D.** Proliferation assay in MDA-MB-231 and BT549 cells. RACK1 overexpressed cells and the corresponding control cells were used. Quantitative analysis was shown. Confluence was analyzed by ImageJ. n=3, biological replicates. **E.** Wound healing assay in MDA-MB-231 and BT549 cells. RACK1 overexpressed cells and the corresponding control cells were used. Quantitative analysis was shown. Relative wound density was determined by IncuCyte. n=3, biological replicates.

**Figure S7** CCDC102B activates NF‑κB pathway. **A.** and **B.** Representative IHC images of p65 in orthotopic xenograft tumors and metastasis lungs in nude mice. (n=12 of each group in orthotopic xenograft breast cancer model; n=6 of each group in tail vein model) Scale bars: 100 μm. **C.** Cell functional rescue tests with Transwell assay in MDA-MB-231 cells. RACK1 overexpressed cells, NF‑κB activated cells and the corresponding control cells were used. Representative photos and quantitative analysis were shown. Confluence of Transwell was analyzed by ImageJ. n=3, biological replicates. Scale bars: 100 μm. **D.** Overexpressed RACK1 was associated with decreased p65. **E.** IP assay of interaction between CCDC102B and IKKα.

**Table S1** Primers sequences for PCR

**Table S2** Oligonucleotide sequences for CRISPR/Cas9 editing

**Table S3** The sgRNA oligos of CCDC102B and RACK1

**Table S4** Spinfection test with different volumes of virus in order to achieve a MOI of 0.3

**Table S5** Antibodies used in the study

**Table S6** Patient characteristics of gene microarray

**Table S7** MAGeCK analysis of high throughput sequencing in three lungs which received the sgRNA library and pre-injection control cells

**Table S8** The 20 targets for which 5 sgRNAs were enriched less than control

**Table S9** Top 20 genes that were least enriched in metastasis lungs compared to initial cell pool according to MAGeCK analysis

**Table S10** Merged results of Supplementary Table 8 and Supplementary Table 9

**Table S11** Clinicopathological characteristics of breast cancer patients in tissue microarray and correlation with CCDC102B expression

**Table S12** Multivariate Cox regression model analysis of independent predictive factors of RFS

**Table S13** Multivariate Cox regression model analysis of independent predictive factors of OS

**Table S14** Results of mass spectrometry (MS) assay showing potential interaction partners binding to CCDC102B

**Table S15** Positive signaling pathways of GSEA in CCDC102B overexpression MDA-MB-231

**Table S16** Negative signaling pathways of GSEA in CCDC102B overexpression MDA-MB-231
