# Supplementary material for: Stabilization of CCDC102B by Loss of RACK1 Through the CMA Pathway Promotes Breast Cancer Metastasis via Activation of the NF-κB Pathway
Source: Front Oncol. 2022 Jul 25;12:927358. doi: 10.3389/fonc.2022.927358 (PMC9359432; doi:10.3389/fonc.2022.927358)
Supplement: Supplementary file 1 [file DataSheet_1.zip › supplementary/Supplementary Table 9 Top 20 genes that were least enriched in metastasis lungs compared to initial cell pool according to MAGeCK analysis.docx]

| Top 20 | Lung 1 vs Control | Lung 2 vs Control | Lung 3 vs Control |
| --- | --- | --- | --- |
| 1 | KCNA3 | SLAMF1 | CD79B |
| 2 | CORO1A | PROX1 | UBASH3A |
| 3 | CD79A | CORO1A | IL2RG |
| 4 | SLAMF6 | CD27 | SP4 |
| 5 | CD28 | CLEC4M | FDCSP |
| 6 | TREML2 | CETP | SPOCK2 |
| 7 | P2RY10 | MARCO | C16orf54 |
| 8 | TMEM71 | TMC8 | GIMAP1-GIMAP5 |
| 9 | LOC256021 | STAP1 | FLT3 |
| 10 | CYTH1 | CD5L | BACH2 |
| 11 | ITGA8 | C16orf54 | ITK |
| 12 | JAK3 | SELL | SP140 |
| 13 | PLCG2 | ST6GAL1 | LRRN3 |
| 14 | CCDC102B | SMAP2 | STAB2 |
| 15 | MMRN1 | JAK3 | FAM65B |
| 16 | PSIP1 | BLNK | WDFY4 |
| 17 | CD5L | CD19 | FCRL2 |
| 18 | PPM1K | GCSAM | TSPAN7 |
| 19 | PLA1A | RHOF | ATF7IP2 |
| 20 | ABCD2 | CCDC102B | CD6 |

Supplementary Table 9 Top 20 genes that were least enriched in metastasis lungs compared to initial cell pool according to MAGeCK analysis
