# Supplementary material for: Stabilization of CCDC102B by Loss of RACK1 Through the CMA Pathway Promotes Breast Cancer Metastasis via Activation of the NF-κB Pathway
Source: Front Oncol. 2022 Jul 25;12:927358. doi: 10.3389/fonc.2022.927358 (PMC9359432; doi:10.3389/fonc.2022.927358)
Supplement: Supplementary file 1 [file DataSheet_1.zip › supplementary/Supplementary Table 13 Multivariate Cox regression model analysis of independent predictive factors of OS.docx]

Supplementary Table 13 Multivariate Cox regression model analysis of independent predictive factors of OS

| Variables | Value | *P* value, OR (95% CI) |
| --- | --- | --- |
| Age | >50 vs ≤50 | 0.182, 2.366(0.668-8.379) |
| Tumor size (pT) | >2cm（pT2-3）vs ≤2cm（pT1） | 0.351, 2.969(0.301-29.290) |
| LN status (pN) | Positive (pN1-3) vs Negative (pN0) | 0.576, 1.555(0.332-7.294) |
| Grade | III vs I-II | 0.886, 1.091(0.330-3.605) |
| LVI | Positive vs Negative | 0.436, 1.742(0.431-7.030) |
| ER | Positive vs Negative | 0.014, 0.117(0.021-0.647) |
| HER2 | Positive vs Negative | 0.439, 0.621(0.186-2.076) |
| CCDC102B | High expression vs Low expression | 0.002, 9.112(2.276-36.484) |

Abbreviations: OS, overall survival; OR, odd ratio; CI, confidential interval; LN, lymph node; LVI, lymphovascular invasion; ER, estrogen receptor; HER2, human epidermal growth factor receptor 2
