# Supplementary material for: Stabilization of CCDC102B by Loss of RACK1 Through the CMA Pathway Promotes Breast Cancer Metastasis via Activation of the NF-κB Pathway
Source: Front Oncol. 2022 Jul 25;12:927358. doi: 10.3389/fonc.2022.927358 (PMC9359432; doi:10.3389/fonc.2022.927358)
Supplement: Supplementary file 1 [file DataSheet_1.zip › supplementary/Supplementary Table 12 Multivariate Cox regression model analysis of independent predictive factors of RFS.docx]

Supplementary Table 12 Multivariate Cox regression model analysis of independent predictive factors of RFS

| Variables | Value | *P* value, OR (95% CI) |
| --- | --- | --- |
| Age | >50 vs ≤50 | 0.699, 1.151(0.564-2.346) |
| Tumor size (pT) | >2cm（pT2-3）vs ≤2cm（pT1） | 0.262, 2.032(0.589-7.005) |
| LN status (pN) | Positive (pN1-3) vs Negative (pN0) | 0.398, 1.525(0.573-4.058) |
| Grade | III vs I-II | 0.583, 0.806(0.373-1.742) |
| LVI | Positive vs Negative | 0.391, 1.460(0.614-3.470) |
| ER | Positive vs Negative | 0.172, 0.578(0.264-1.269) |
| HER2 | Positive vs Negative | 0.639, 0.838(0.401-1.753) |
| CCDC102B | High expression vs Low expression | 0.009, 2.672(1.284-5.563) |

Abbreviations: RFS, recurrence-free survival; OR, odd ratio; CI, confidential interval; LN, lymph node; LVI, lymphovascular invasion; ER, estrogen receptor; HER2, human epidermal growth factor receptor 2
