# Supplementary material for: Stabilization of CCDC102B by Loss of RACK1 Through the CMA Pathway Promotes Breast Cancer Metastasis via Activation of the NF-κB Pathway
Source: Front Oncol. 2022 Jul 25;12:927358. doi: 10.3389/fonc.2022.927358 (PMC9359432; doi:10.3389/fonc.2022.927358)
Supplement: Supplementary file 1 [file DataSheet_1.zip › supplementary/Supplementary Table 1 Primers sequences for PCR-UPDATE.docx]

Supplementary Table 1 Primers sequences for PCR.

CCDC102B-F1: CCGGTGAAAGTTATGCTGAGGG

CCDC102B-R1: GACAAAGCCGACTCCAGTCTCTC

CCDC102B-F2: CAGCTTTGCAGGTGCATTTGGATG

CCDC102B-R2: CCTGCCATCCCCACCACATAGAC

CCDC102B-F: CTAGCTAGCTAGCCACCATGAATTTAGATTCCATACATCGATTAATTG

CCDC102B-R: CGGGATCCCGACCAGTTTTGCAAGTGCCTGAG

RACK1-F: CTAGCTAGCTAGATGACTGAGCAGATGACCCTTCG

RACK1-R: ATTTGCGGCCGCTTTAGCACAGTCGAGGCTGATCAGCGG

RACK1-QPCR-F: AGCAGCAACCCTATCATCGTC

RACK1-QPCR-R: TGAGATCCCATAACATGGCCT

CCDC102B-QPCR-F: AGCTTTGCAGGTGCATTTGG

CCDC102B-QPCR-R: TCCACAGAGACAAAGCCGAC

ACTB-F: CTCCATCCTGGCCTCGCTGT

ACTB-R: GCTGTCACCTTCACCGTTCC

humans-specific-F：ATGCTGATGTCTGGGTAGGGTG

humans-specific-R：TGAGTCAGGAGCCAGCGTATG

deep sequencing STEP1-F：AATGGACTATCATATGCTTACCGTAACTTGAAAGTATTTCG

deep sequencing STEP1-R：CTTTAGTTTGTATGTCTGTTGCTATTATGTCTACTATTCTTTCC

deep sequencing STEP2-F：AATGATACGGCGACCACCGAGATCTACACTCTTTCCCTACACGACGCTCTTCCGATCTTCTTGTGGAAAGGACGAAACACCG

deep sequencing STEP2-R：CAAGCAGAAGACGGCATACGAGATAAGTAGAGGTGACTGGAGTTCAGACGTGTGCTCTTCCGATCTTTCTACTATTCTTTCCCCTGCACTGT

CCDC102B-SHORT-F: CTAGCTAGCCACCATGAATTTAG

CCDC102B-SHORT-A-R: CGCGGATCCGCGACTCTGTTTCTTTTTCAGTGTACTC

CCDC102B-SHORT-B-R: CGCGGATCCGCGAGATTTGGATTCTTCCTTTATATTGC

CCDC102B-SHORT-C-R: CGCGGATCCGCGCCAGTTTTGCAAGTGCC

CCDC102B-SHORT-C1-R: CGCGGATCCGCGCTCCGAGGTATTTTCAGCTTGC

CCDC102B-SHORT-C2-R: CGCGGATCCGCGTAATCTATTTTTCTTATCCAAAAGCTCTTCC

CCDC102B-SHORT-C3-R: CGCGGATCCGCGCAGGGAATCATCAAGCTCATCTTC

RACK1-SHORT-F: GCTCTAGAGCATGACTGAGCAGATGACCC

RACK1-SHORT-1R: CGGGGTACCCCGATTCCATAGCTTGATGGTTTTATCTCG

RACK1-SHORT-2R: CGGGGTACCCCGGTTCCATACCTTGACCAGCTTG

RACK1-SHORT-3R: CGGGGTACCCCGATCCCATAACATGGCCTGGC

RACK1-SHORT-4R: CGGGGTACCCCGATCCCAGATCTTGATGCTGGG

RACK1-SHORT-5R: CGGGGTACCCCGGCGTGTGCCAATGGTCAC

EGR1-F: CCACGCCGAACACTGACATT

EGR1-R: GAGGGGTTAGCGAAGGCTG

INHBA-F: ACGGGTATGTGGAGATAGAGGA

INHBA-R: GGACTTTTAGGAAGAGCCAGACT

FOS-F: CACTCCAAGCGGAGACAGAC

FOS-R: AGGTCATCAGGGATCTTGCAG

PTGS2-F: TAAGTGCGATTGTACCCGGAC

PTGS2-R: TTTGTAGCCATAGTCAGCATTGT
