# Supplementary material for: Stabilization of CCDC102B by Loss of RACK1 Through the CMA Pathway Promotes Breast Cancer Metastasis via Activation of the NF-κB Pathway
Source: Front Oncol. 2022 Jul 25;12:927358. doi: 10.3389/fonc.2022.927358 (PMC9359432; doi:10.3389/fonc.2022.927358)
Supplement: Supplementary file 1 [file DataSheet_1.zip › supplementary/Supplementary Table 3 The sgRNA oligos of CCDC102B and RACK1.docx]

Supplementary Table 3 The sgRNA oligos of CCDC102B and RACK1

CCDC102B-CRISPR-1-F: CACCGTTTGTGAAGAACTTCGCCTG

CCDC102B-CRISPR-2-F: CACCGTTCCTCAATTAATCGATGTA

CCDC102B-CRISPR-3-F: CACCGGGAAACGGTGAAACGAAAAC

CCDC102B-CRISPR-4-F: CACCGGATGAAATGCAAGAACTGTC

CCDC102B-CRISPR-5-F: CACCGTAAATCAACACCATCTAGAT

CCDC102B-CRISPR-6-F: CACCGGATTTGTGAGTTAAGAGCAG

CCDC102B-CRISPR-1-R: AAACCAGGCGAAGTTCTTCACAAAC

CCDC102B-CRISPR-2-R: AAACTACATCGATTAATTGAGGAAC

CCDC102B-CRISPR-3-R: AAACGTTTTCGTTTCACCGTTTCCC

CCDC102B-CRISPR-4-R: AAACGACAGTTCTTGCATTTCATCC

CCDC102B-CRISPR-5-R: AAACATCTAGATGGTGTTGATTTAC

CCDC102B-CRISPR-6-R: AAACCTGCTCTTAACTCACAAATCC

RACK1-CRISPR-1-F: CACCGATGGCCCACAAATCGCCTCG

RACK1-CRISPR-2-F: CACCGTCCATAGTTGGTCTCATCCC

RACK1-CRISPR-3-F: CACCGAACCCTATCATCGTCTCCTG

RACK1-CRISPR-4-F: CACCGCCGTGTTCAGATAGCCTGTG

RACK1-CRISPR-1-R: AAACCGAGGCGATTTGTGGGCCATC

RACK1-CRISPR-2-R: AAACGGGATGAGACCAACTATGGAC

RACK1-CRISPR-3-R: AAACCAGGAGACGATGATAGGGTTC

RACK1-CRISPR-4-R: AAACCACAGGCTATCTGAACACGGC
