# Supplementary material for: Stabilization of CCDC102B by Loss of RACK1 Through the CMA Pathway Promotes Breast Cancer Metastasis via Activation of the NF-κB Pathway
Source: Front Oncol. 2022 Jul 25;12:927358. doi: 10.3389/fonc.2022.927358 (PMC9359432; doi:10.3389/fonc.2022.927358)
Supplement: Supplementary file 1 [file DataSheet_1.zip › supplementary/Supplementary Table 7 MAGeCK analysis of high throughput sequencing in three lungs which received the sgRNA library and pre-injection control cells.docx]

Supplementary Table 7 MAGeCK analysis of high throughput sequencing in three lungs which received the sgRNA library and pre-injection control cells.

|  | Reads | Mapped | Percentage | TotalsgRNAs | Zerocounts |
| --- | --- | --- | --- | --- | --- |
| Control | 10447484 | 5982210 | 0.5726 | 2000 | 483 |
| Lung 1 | 9279812 | 2083487 | 0.2245 | 2000 | 355 |
| Lung 2 | 9460932 | 2748449 | 0.2905 | 2000 | 370 |
| Lung 3 | 8653870 | 1700414 | 0.1965 | 2000 | 406 |

Zerocounts were missed sgRNAs, which were all nontargeting control sgRNAs for human.
