# Supplementary material for: Stabilization of CCDC102B by Loss of RACK1 Through the CMA Pathway Promotes Breast Cancer Metastasis via Activation of the NF-κB Pathway
Source: Front Oncol. 2022 Jul 25;12:927358. doi: 10.3389/fonc.2022.927358 (PMC9359432; doi:10.3389/fonc.2022.927358)
Supplement: Supplementary file 1 [file DataSheet_1.zip › supplementary/Supplementary Table 14 Results of mass spectrometry (MS) assay showing potential interaction partners binding to CCDC102B.docx]

Supplementary Table 14 Results of mass spectrometry (MS) assay showing potential interaction partners binding to CCDC102B.

| Rank | Reference | PepCount | UniquePepCount | CoverPercent | MW | PI |
| --- | --- | --- | --- | --- | --- | --- |
| 1 | GN=RACK1 PE=1 SV=3 | 19 | 5 | 26.50% | 35076.33 | 7.6 |
| 2 | GN=EIF3F PE=1 SV=1 | 9 | 3 | 14.01% | 37563.42 | 5.24 |
| 3 | GN=GAPDH PE=1 SV=3 | 8 | 3 | 12.84% | 36052.79 | 8.57 |
| 4 | GN=PCBP2 PE=1 SV=1 | 6 | 3 | 16.16% | 38579.66 | 6.33 |
| 5 | GN=SLC25A5 PE=1 SV=7 | 11 | 2 | 9.06% | 32851.84 | 9.71 |
| 6 | GN=PHB2 PE=1 SV=2 | 6 | 2 | 9.70% | 33295.97 | 9.83 |
| 7 | GN=PCBP1 PE=1 SV=2 | 6 | 2 | 12.08% | 37497.43 | 6.66 |
| 8 | GN=SLC25A6 PE=1 SV=4 | 5 | 2 | 9.06% | 32865.87 | 9.76 |
| 9 | GN=VDAC1 PE=1 SV=2 | 5 | 2 | 10.60% | 30772.21 | 8.62 |
| 10 | GN=RPLP0 PE=1 SV=1 | 5 | 2 | 9.46% | 34273.11 | 5.72 |
| 11 | GN=RPS2 PE=1 SV=2 | 3 | 2 | 9.90% | 31324.07 | 10.25 |

MW 30000-40000 Unique pep count > 1
