# Supplementary figures and images for: Stabilization of CCDC102B by Loss of RACK1 Through the CMA Pathway Promotes Breast Cancer Metastasis via Activation of the NF-κB Pathway
Source: Front Oncol. 2022 Jul 25;12:927358. doi: 10.3389/fonc.2022.927358 (PMC9359432; doi:10.3389/fonc.2022.927358)

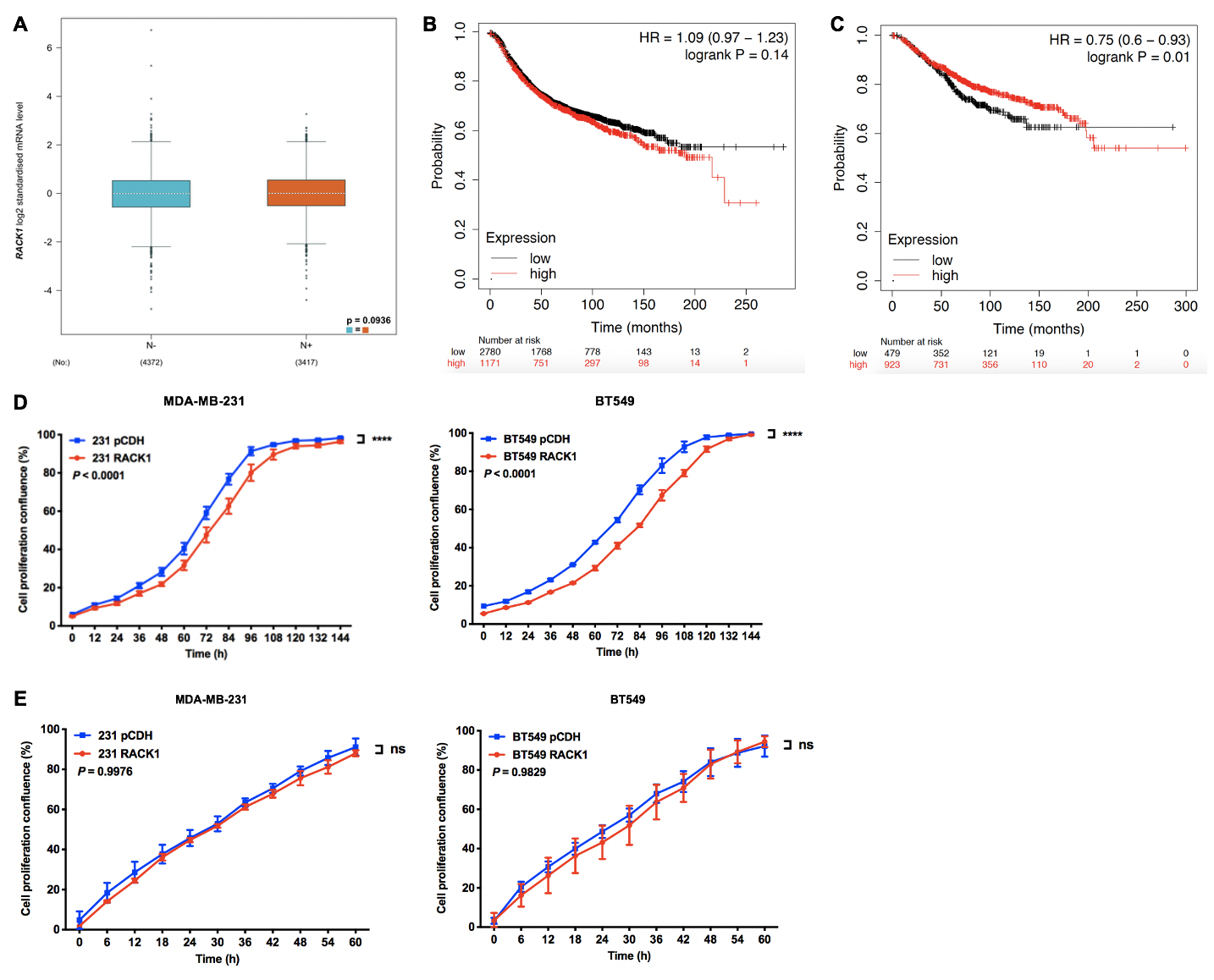

Supplement: Supplementary file 1 [file DataSheet_1.zip › supplementary/Supplementary figure 6.png]

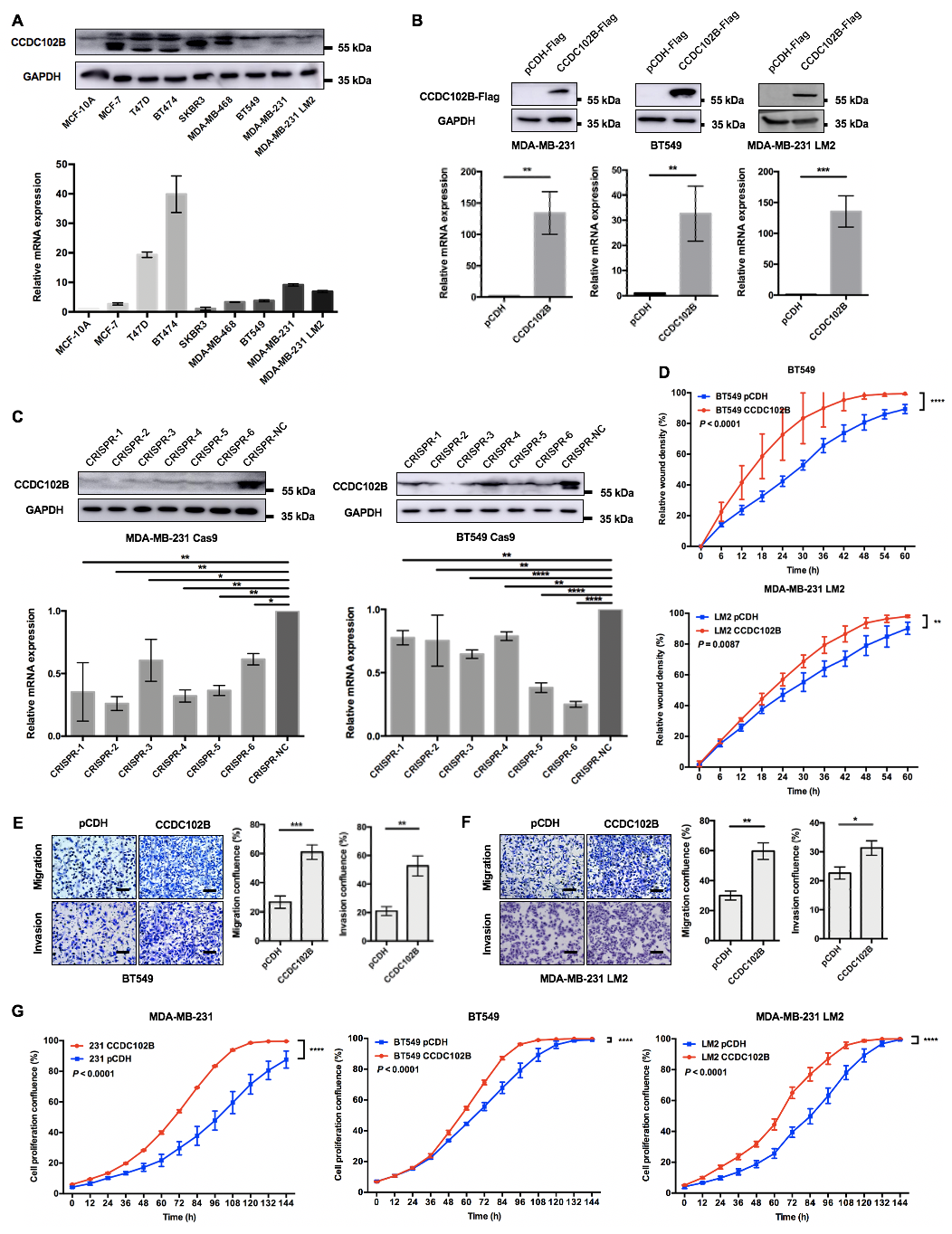

Supplement: Supplementary file 1 [file DataSheet_1.zip › supplementary/Supplementary figure 3-1.png]

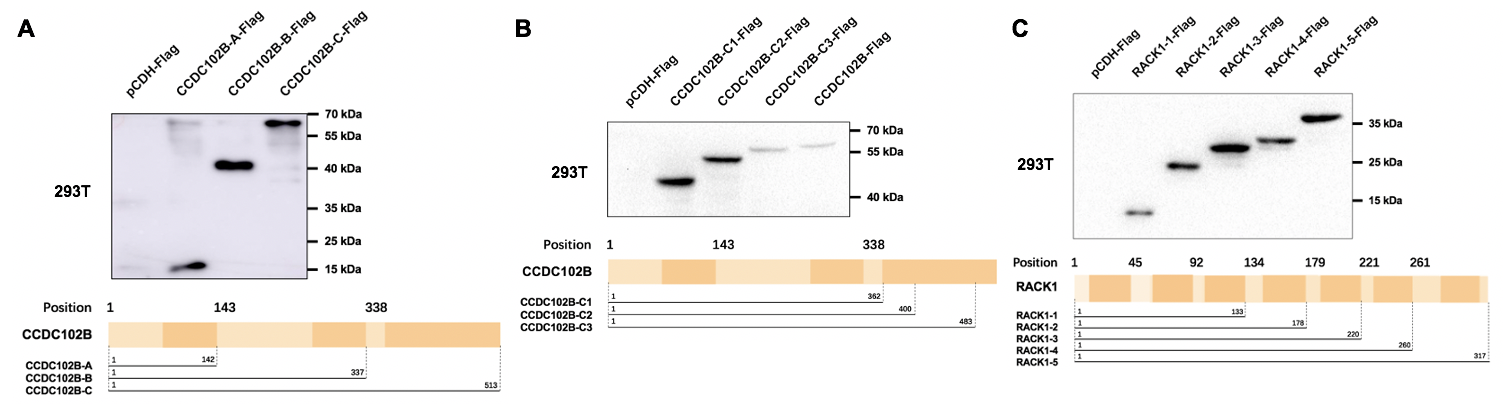

Supplement: Supplementary file 1 [file DataSheet_1.zip › supplementary/Supplementary figure 5.png]

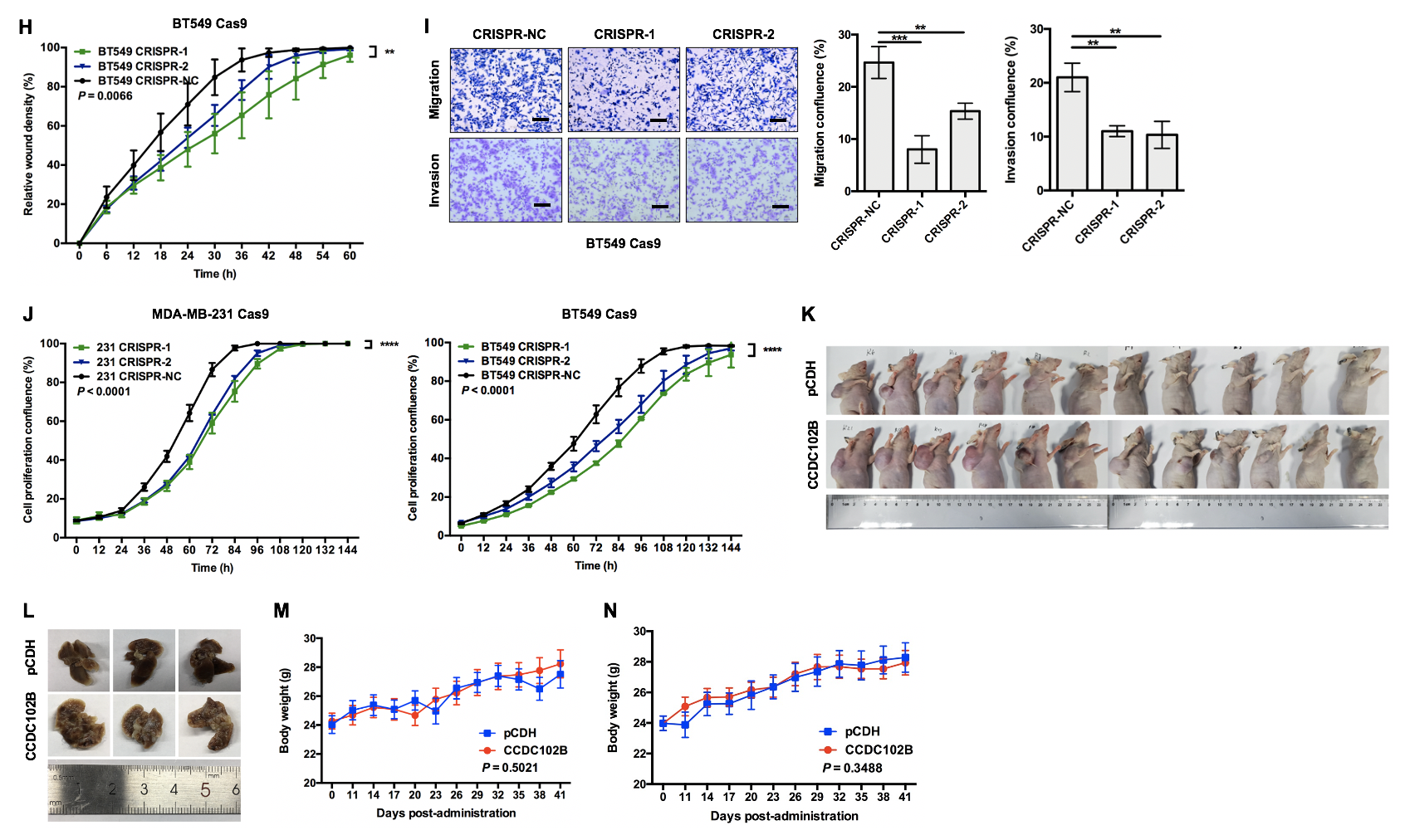

Supplement: Supplementary file 1 [file DataSheet_1.zip › supplementary/Supplementary figure 3-2.png]

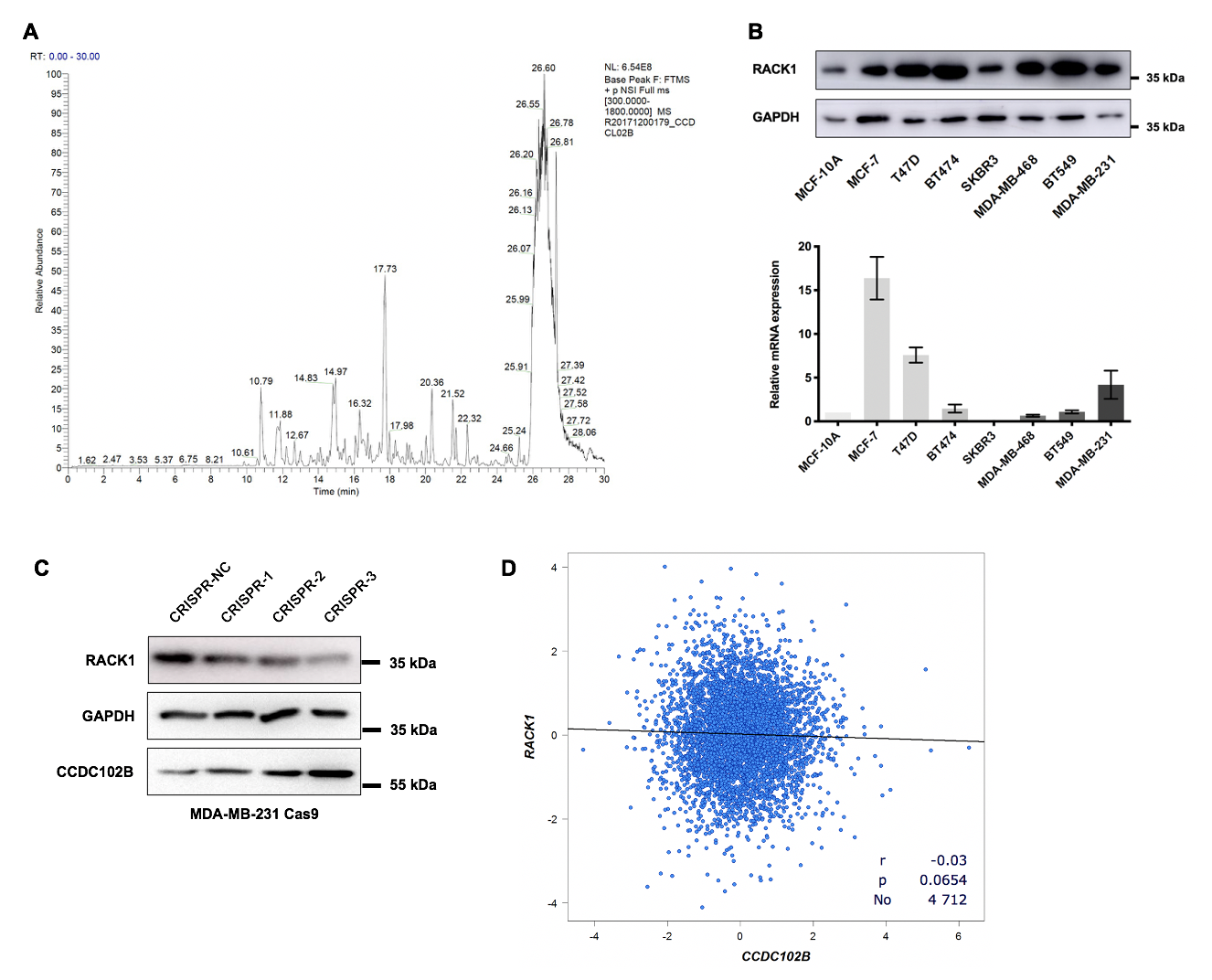

Supplement: Supplementary file 1 [file DataSheet_1.zip › supplementary/Supplementary figure 4.png]

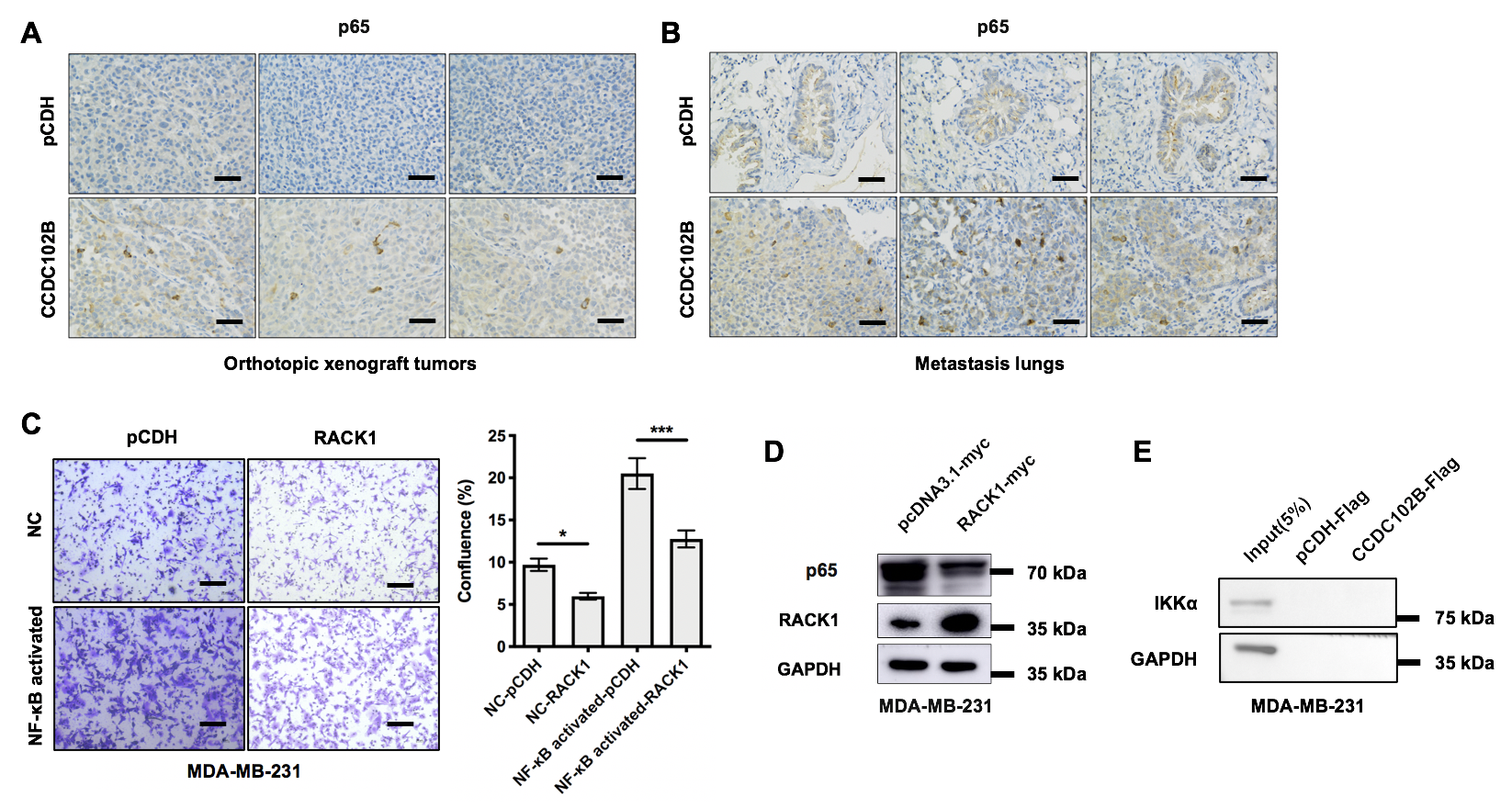

Supplement: Supplementary file 1 [file DataSheet_1.zip › supplementary/Supplementary figure 7-UPDATE.png]

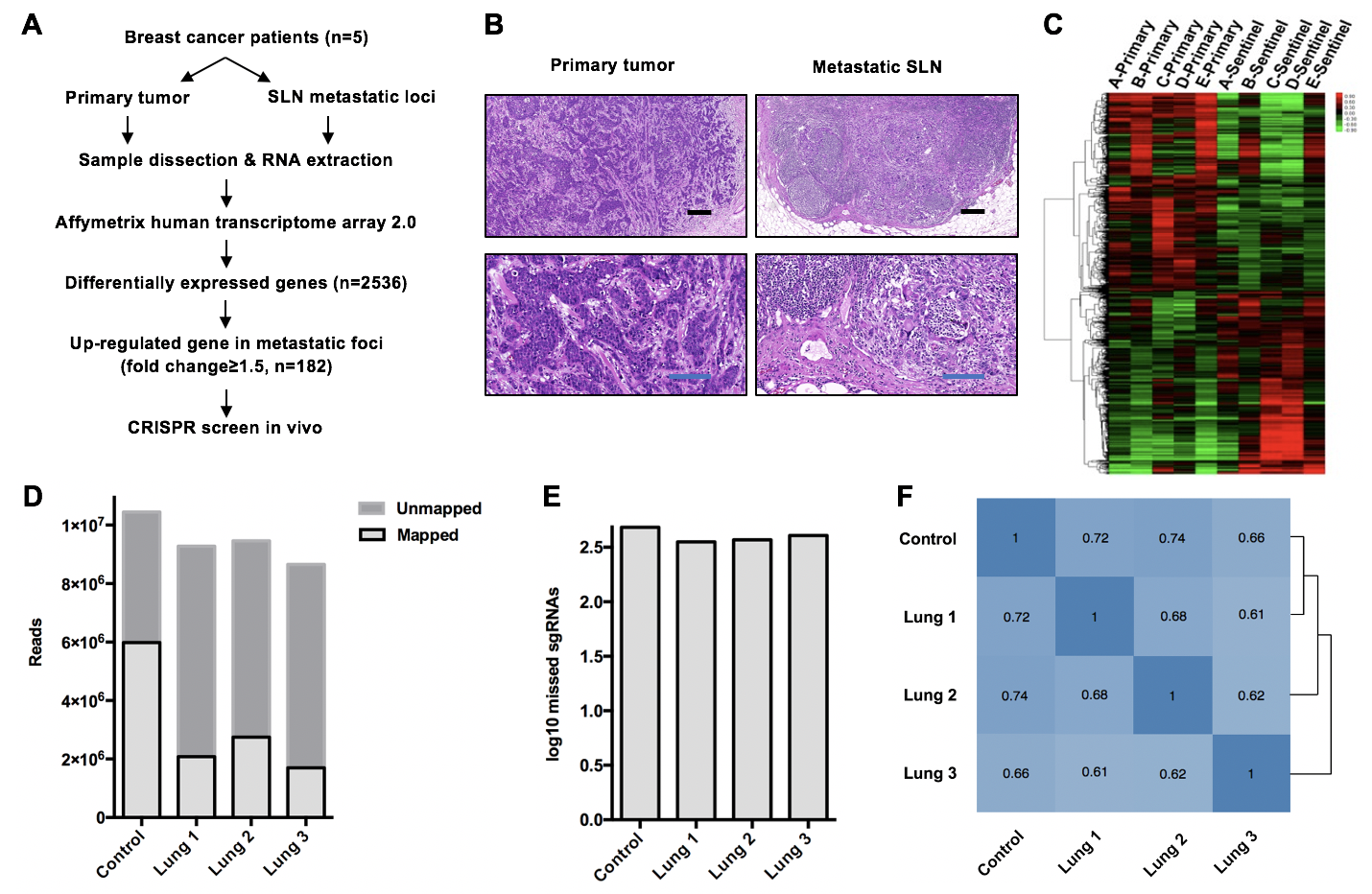

Supplement: Supplementary file 1 [file DataSheet_1.zip › supplementary/Supplementary figure 1.png]

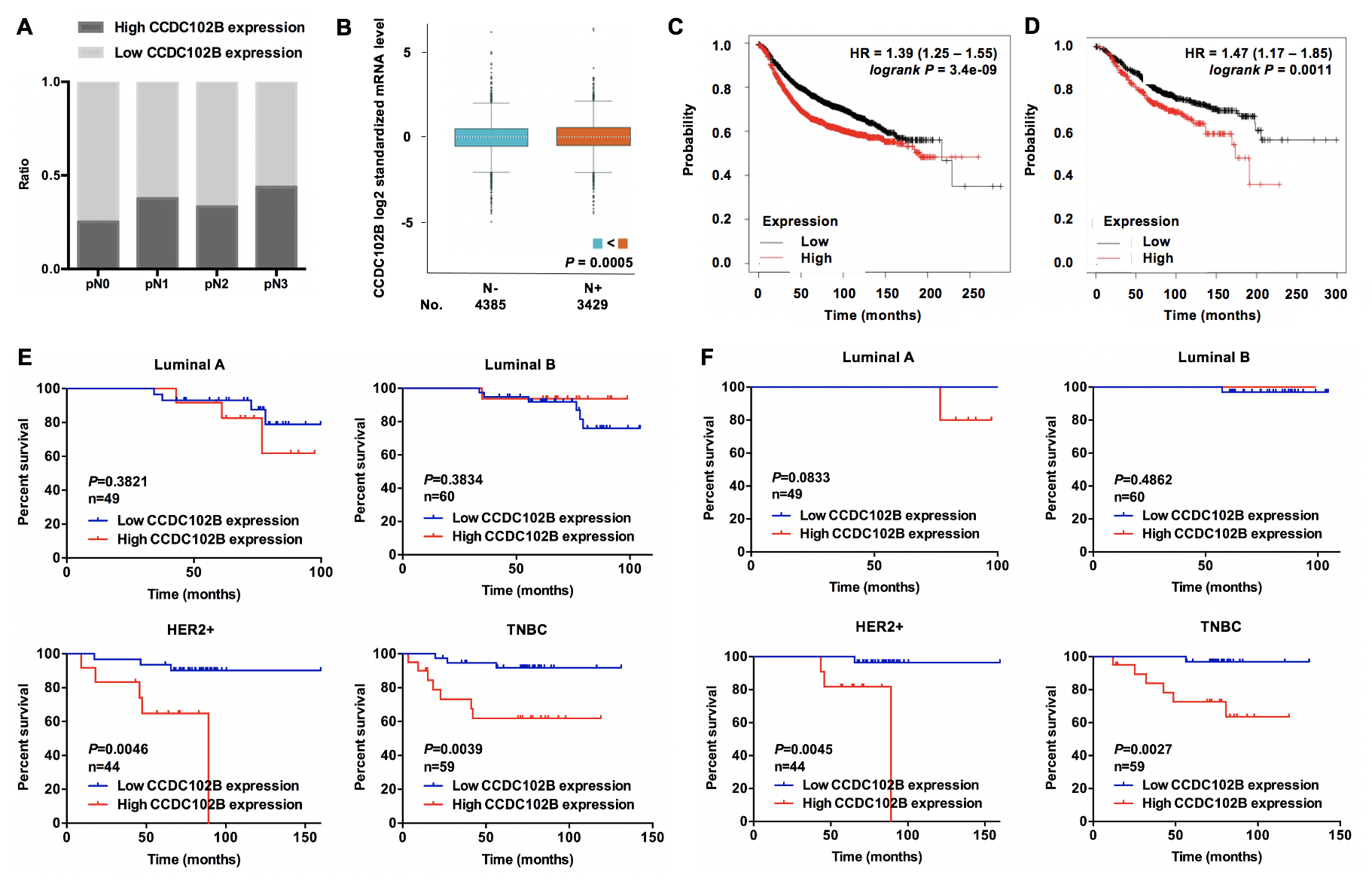

Supplement: Supplementary file 1 [file DataSheet_1.zip › supplementary/Supplementary figure 2.png]
